# Two di­alkyl­ammonium salts of 2-amino-4-nitro­benzoic acid: crystal structures and Hirshfeld surface analysis

**DOI:** 10.1107/S2056989016017266

**Published:** 2016-11-01

**Authors:** James L. Wardell, Mukesh M. Jotani, Edward R. T. Tiekink

**Affiliations:** a Fundaçaö Oswaldo Cruz, Instituto de Tecnologia em Fármacos-Far Manguinhos, 21041-250 Rio de Janeiro, RJ, Brazil; b Department of Chemistry, University of Aberdeen, Old Aberdeen AB24 3UE, Scotland; c Department of Physics, Bhavan’s Sheth R. A. College of Science, Ahmedabad, Gujarat 380001, India; d Research Centre for Crystalline Materials, Faculty of Science and Technology, Sunway University, 47500 Bandar Sunway, Selangor Darul Ehsan, Malaysia

**Keywords:** crystal structure, carboxyl­ate, salt, hydrogen bonding, Hirshfeld surface analysis

## Abstract

The crystal structures of two ammonium salts of 2-amino-4-nitro­benzoic acid are described, namely di­methyl­aza­nium 2-amino-4-nitro­benzoate, C_2_H_8_N^+^·C_7_H_5_N_2_O_4_^−^, (I), and di­butyl­aza­nium 2-amino-4-nitro­benzoate, C_8_H_20_N^+^·C_7_H_5_N_2_O_4_^−^, (II). The asymmetric unit of (I) comprises a single cation and a single anion. In the anion, small twists are noted for the carboxyl­ate and nitro groups from the ring to which they are connected, as indicated by the dihedral angles of 11.45 (13) and 3.71 (15)°, respectively; the dihedral angle between the substituents is 7.9 (2)°. The asymmetric unit of (II) comprises two independent pairs of cations and anions. In the cations, different conformations are noted in the side chains in that three chains have an all-*trans* [(+)-anti­periplanar] conformation, while one has a distinctive kink resulting in a (+)-synclinal conformation. The anions, again, exhibit twists with the dihedral angles between the carboxyl­ate and nitro groups and the ring being 12.73 (6) and 4.30 (10)°, respectively, for the first anion and 8.1 (4) and 12.6 (3)°, respectively, for the second. The difference between anions in (I) and (II) is that in the anions of (II), the terminal groups are conrotatory, forming dihedral angles of 17.02 (8) and 19.0 (5)°, respectively. In each independent anion of (I) and (II), an intra­molecular amino-N—H⋯O(carboxyl­ate) hydrogen bond is formed. In the crystal of (I), anions are linked into a jagged supra­molecular chain by charge-assisted amine-N—H⋯O(carboxyl­ate) hydrogen bonds and these are connected into layers *via* charge-assisted ammonium-N—H⋯O(carboxyl­ate) hydrogen bonds. The resulting layers stack along the *a* axis, being connected by nitro-N—O⋯π(arene) and methyl-C—H⋯O(nitro) inter­actions. In the crystal of (II), the anions are connected into four-ion aggregates by charge-assisted amino-N—H⋯O(carboxyl­ate) hydrogen bonding. The formation of ammonium-N—H⋯O(carboxyl­ate) hydrogen bonds, involving all ammonium-N—H and carboxyl­ate O atoms leads to a three-dimensional architecture; additional C—H⋯O(nitro) inter­actions contribute to the packing. The Hirshfeld surface analysis confirms the importance of the hydrogen bonding in both crystal structures. Indeed, O⋯H/H⋯O inter­actions contribute nearly 50% to the entire Hirshfeld surface in (I).

## Abstract 2

The structures of two ammonium salts of 2-amino-4-nitro­benzoic acid are described. Substantial hydrogen bonding leads to supra­molecular layers in the [Me_2_NH_2_]^+^ salt and a three-dimensional architecture in the case of the [*n*-Bu_2_NH_2_]^+^ salt.

## Chemical context   

The simple carb­oxy­lic acid 2-amino-4-nitro­benzoic acid has been little studied from a crystallographic point of view: its mol­ecular structure was only reported in 2011 (Wardell & Tiekink, 2011 ▸). Very recently, a number of polymorphs were described, *i.e*. with *Z*′ = 1, 2 and 3 (Wardell & Wardell, 2016 ▸). The only other two structures described with 2-amino-4-nitro­benzoic acid are its 2:1 co-crystal with 2,2′-bipyridyl and its 1:1 co-crystal with bis­(pyridin-2-yl)methanone (Wardell & Tiekink, 2011 ▸). Besides the structure of a Pb^II^ coordination polymer (Chen & Huang, 2009 ▸), the remaining literature structures are salts featuring 2-amino-4-nitro­benzoic acid in its mono-anionic form, exclusively with a deprotonated carboxyl­ate group. Thus, the structures of alkali metal salts, *i.e*. Na^+^, K^+^ (Smith, 2013 ▸), Rb^+^ (Smith, 2014*a* ▸) and Cs^+^ (Smith & Wermuth, 2011 ▸) have been described along with a number of ammonium salts, *i.e*. with NH_4_, as a hydrate (Smith, 2014*b* ▸), di­cyclo­hexyl­ammonium (Smith *et al.*, 2004 ▸), guanidinium, as a hydrate (Smith *et al.*, 2007 ▸), morpholinium (Smith & Lynch, 2016 ▸) and ethyl­enedi­ammonium, as a dihydrate (Smith *et al.*, 2002 ▸). As a continuation of our work in the area noted above (Wardell & Tiekink, 2011 ▸; Wardell & Wardell, 2016 ▸), we describe herein the crystal and mol­ecular structures of two new anhydrous salts of 2-amino-4-nitro­benzoate, with the counter-cations [Me_2_NH_2_]^+^ (I) and [*n*-Bu_2_NH_2_]^+^ (II). Further insight into the self-assembly of the salts has been gained through a Hirshfeld surface analysis.
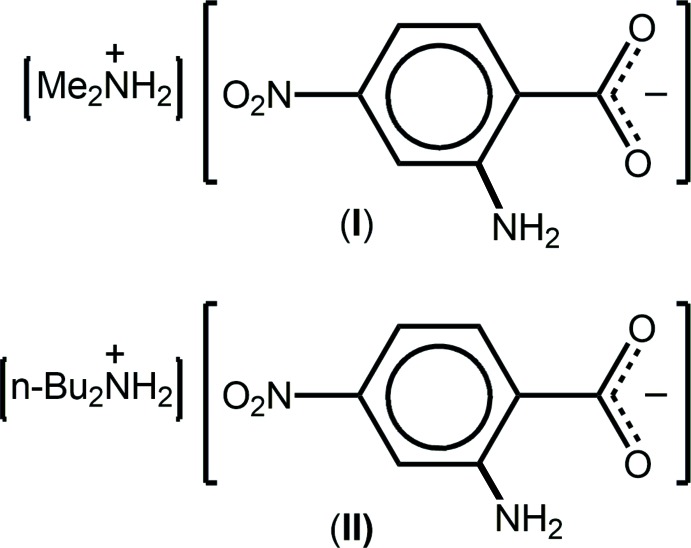


## Structural commentary   

**Figure 1:**
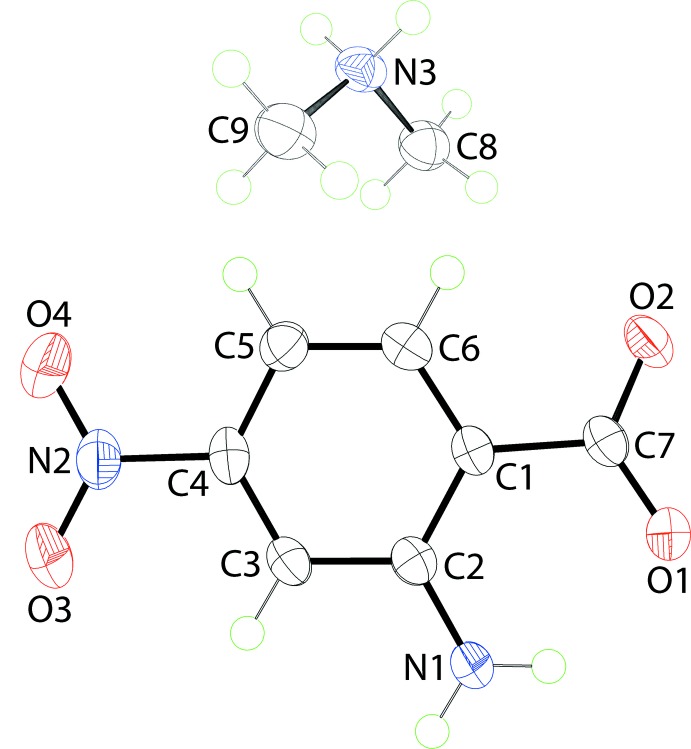
The mol­ecular structure of the constituents of (I), showing the atom-labelling scheme and displacement ellipsoids at the 50% probability level.

The mol­ecular structures of the constituents of (I) are shown in Fig. 1 ▸; the asymmetric unit comprises one cation and one anion. Confirmation of proton transfer during recrystallization of di­methyl­amine and 2-amino-4-nitro­benzoic acid is found in (i) the similarity of the C—O bond lengths [C7—O1, O2 = 1.2587 (17) and 1.2609 (16) Å, respectively] and (ii) the pattern of hydrogen bonding as discussed in *Supra­molecular features*. The mol­ecular structure of the cation is unremarkable with a C8—N3—C9 angle of 113.54 (11)°. The anion features an intra­molecular amino-N—H⋯O(carboxyl­ate) hydrogen bond (Table 1 ▸). Despite the presence of this inter­action, there are small twists in the mol­ecule as seen in the values of the C2—C1—C7—O2 and O3—N2—C4—C3 torsion angles of 169.51 (12) and 4.04 (19)°, respectively. In terms of dihedral angles, the angles between the central ring and the carboxyl­ate and nitro groups are 11.45 (13) and 3.71 (15)°, respectively. The carboxyl­ate and nitro substituents are in the same relative orientation with the dihedral angle between them being 7.9 (2)°.

**Figure 2:**
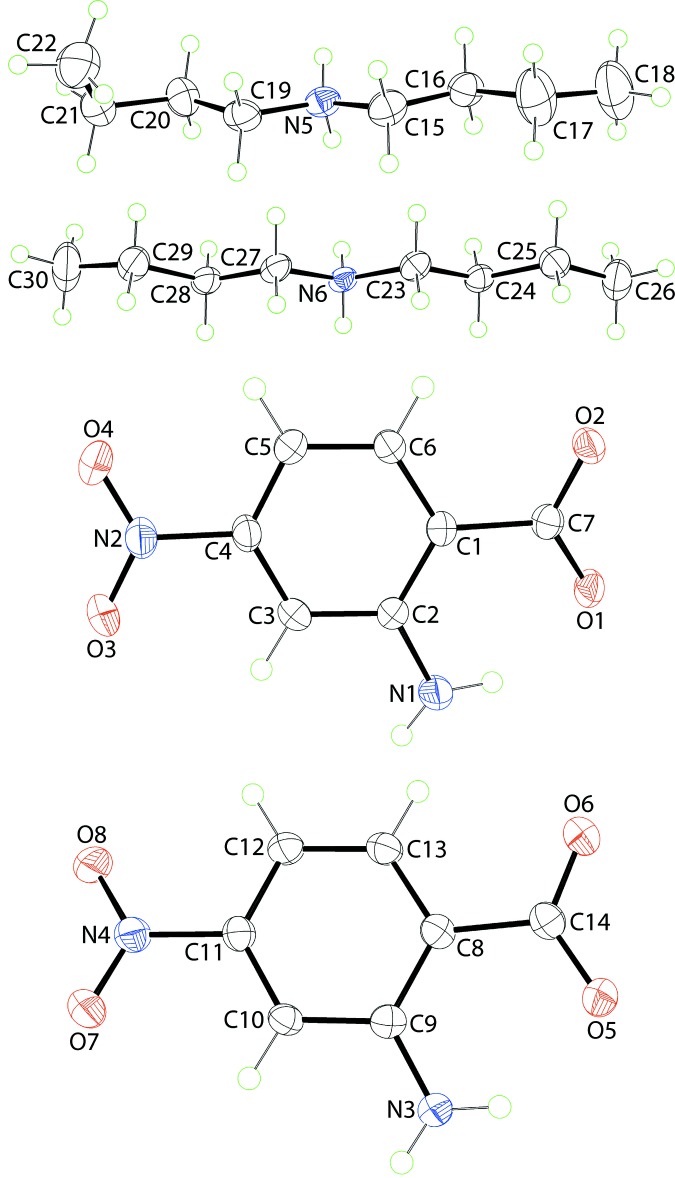
The mol­ecular structure of the constituents of (II), showing the atom-labelling scheme and displacement ellipsoids at the 50% probability level.

The asymmetric unit of (II) comprises two independent pairs of cations and anions. The mol­ecular structures of these are shown Fig. 2 ▸. As for (I), the confirmation for proton transfer from acid to base is seen in the equivalence of the C—O [C7—O1, O2 = 1.262 (2) and 1.267 (3) Å, respectively and C14—O5, O6 = 1.269 (3) and 1.256 (3) Å, respectively] bond lengths and in the pattern of inter­molecular inter­actions, see below. The C15—N5—C19 and C23—N6—C27 angles in the cations are 113.40 (19) and 112.99 (17)°, respectively, *i.e*. similar to the comparable angle in (I). The cations adopt different conformations as seen in the relative orientations of the terminal methyl groups. For the N5-cation, this is qu­anti­fied in the values of the C15—C16—C17—C18 and C19—C20—C21—C22 torsion angles of 171.9 (3) and 49.5 (3)°, respectively, consistent with a (+)-anti­periplanar (+ap) and a (+)-synclinal (+sc) conformation, respectively. In the N6-cation, each chain is +ap, *i.e*. with torsion angles of 173.0 (2)° (C23-chain) and 176.0 (2)° (C27-chain). The anions present similar conformations as in (I) and each features an intra­molecular amino-N—H⋯O(carboxyl­ate) hydrogen bond, Table 2 ▸. However, there are some subtle differences between the anions in terms of the relationship between the central rings and terminal substituents. For the O1-anion, the angles between the central ring and the carboxyl­ate and nitro groups are 12.73 (6) and 4.30 (10)°, respectively, and the comparable angles for the O5-cation are 8.1 (4) and 12.6 (3)°, respectively. The difference between (I) and (II) is that in the cations of (II), the terminal groups are con-rotatory, forming dihedral angles of 17.02 (8) and 19.0 (5)°, respectively.

## Supra­molecular features   

**Figure 3:**
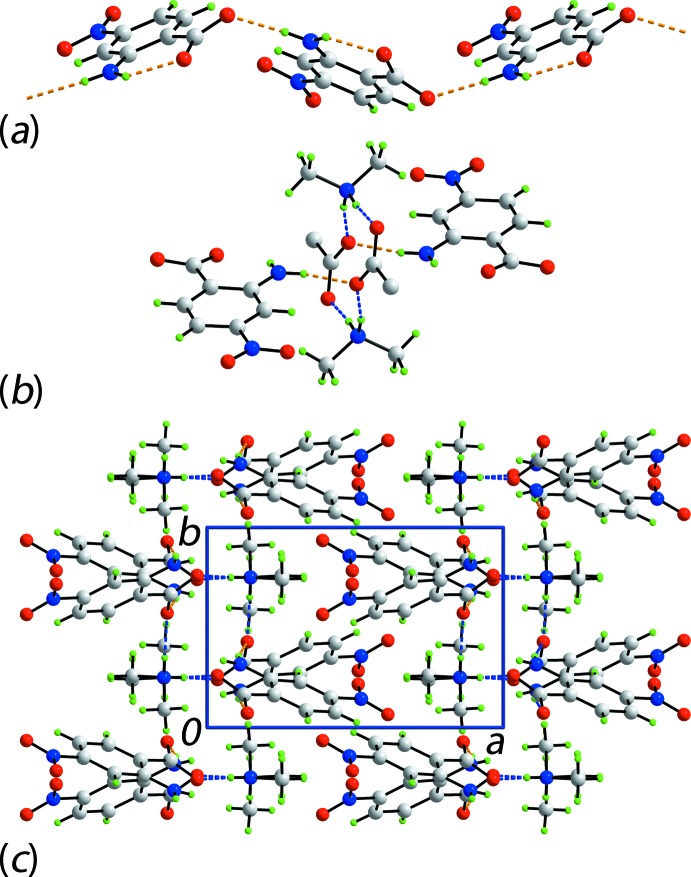
The mol­ecular packing in (I), showing (*a*) supra­molecular chain comprising anions only, orientated along the *c* axis and sustained by amino-N—H⋯O(carboxyl­ate) inter­actions shown as orange dashed lines, (*b*) detail of the 12-membered {⋯HNH⋯OCO}_2_ synthon with ammonium-N—H⋯O(carboxyl­ate) hydrogen bonds shown as blue dashed lines and (*c*) a view of the unit-cell contents in projection down the *c* axis. In part (*b*), all but the CO_2_ groups of the two central benzoate residues have been removed for clarity.

As mentioned above, one H atom of the amino group forms an intra­molecular hydrogen bond with the carboxyl­ate-O1 atom. The second amino-H atom in (I) forms an inter­molecular, charge-assisted amino-N—H⋯O2(carboxyl­ate) hydrogen bond to link anions into a supra­molecular chain, with a jagged topology, aligned along the *c* axis, Fig. 3 ▸ *a*. Translationally-related chains stack along the *a* axis to define cavities in which reside the [Me_2_NH_2_]^+^ cations. These serve to link the anionic chains into layers *via* charge-assisted ammonium-N—H⋯O(carboxyl­ate) hydrogen bonds, involving both carboxyl­ate-O atoms. This association leads to the formation of centrosymmetric, 12-membered {⋯HNH⋯OCO}_2_ synthons, Fig. 3 ▸ *b*. Layers stack along the *a* axis with the most notable inter­actions between the layers being nitro-N—O⋯π(arene) and methyl-C—H⋯O(nitro) contacts. The nitro-O4 atom is crucial in the formation of these contacts, being the donor and acceptor, respectively, Table 1 ▸, Fig. 3 ▸ *c*.

**Figure 4:**
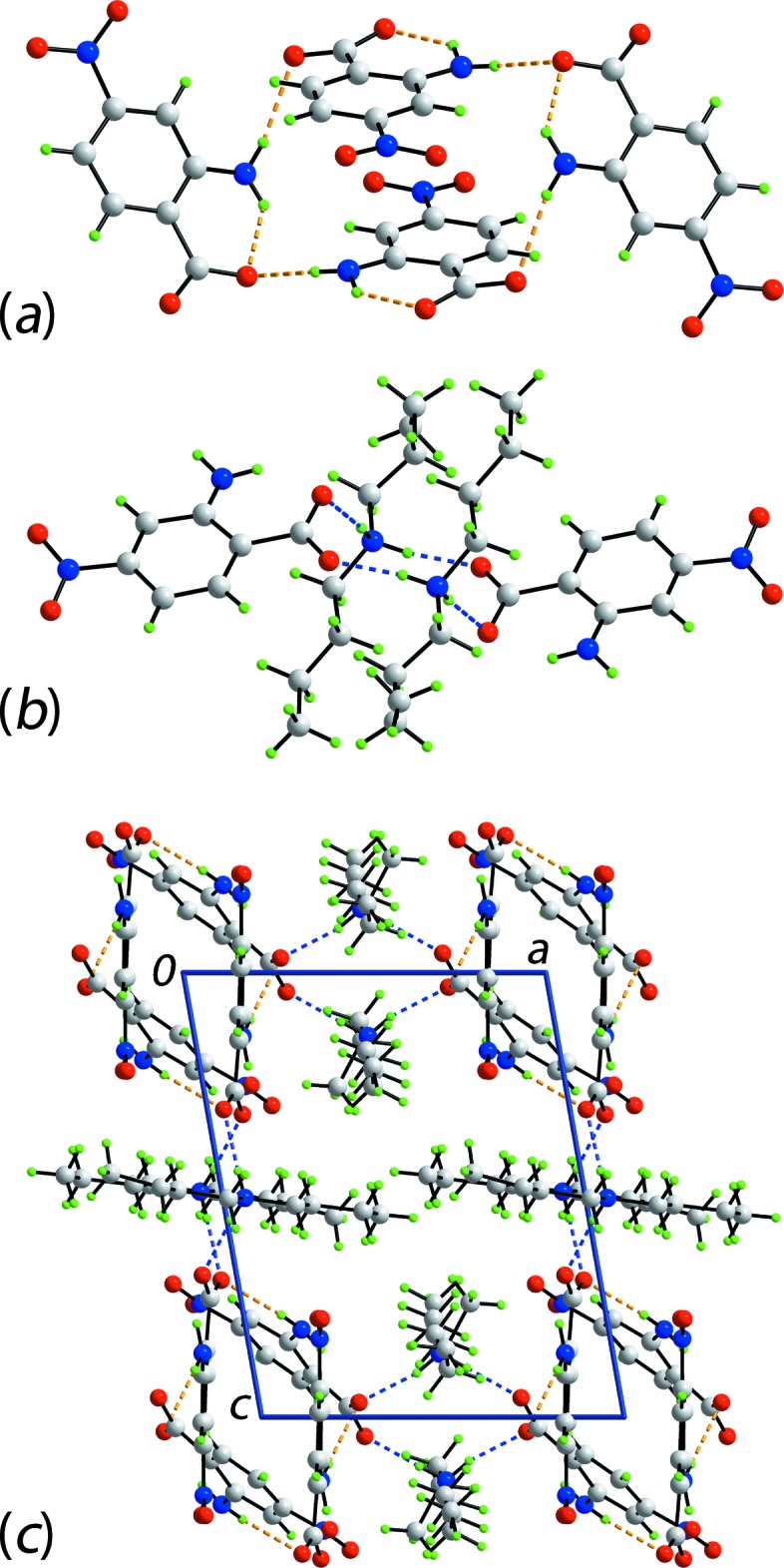
The mol­ecular packing in (II), showing (*a*) four-anion aggregate sustained by amino-N—H⋯O(carboxyl­ate) inter­actions shown as orange dashed lines, (*b*) detail of the 12-membered {⋯HNH⋯OCO}_2_ synthon with ammonium-N—H⋯O(carboxyl­ate) hydrogen bonds shown as blue dashed lines and (*c*) a view of the unit-cell contents in projection down the *b* axis.

The crystal of (II) features extensive N—H⋯O hydrogen bonding, Table 2 ▸. The anions assemble into four-ion aggregates as a result of charge-assisted amino-N—H⋯O(carboxyl­ate) hydrogen bonding. For the O1-anion, the carboxyl­ate-O atom not participating in the intra­molecular amino-N—H⋯O inter­action forms an inter­molecular amino-N—H⋯O inter­action. However, for the O5-anion, the carboxyl­ate-O atom participating in the intra­molecular amino-N—H⋯O inter­action also forms the inter­molecular amino-N—H⋯O contact, as illustrated in Fig. 4 ▸ *a*. The result of this self-assembly is a centrosymmetric, 20-membered {⋯HNH⋯OCO⋯HNH⋯O}_2_ ring which encompasses two {⋯HNC_3_O} loops formed by the intra­molecular amino-N—H⋯O(carboxyl­ate) hydrogen bonds. Each of the cations associates with two anions in a very similar fashion to that in (I), in that the H atoms of the N5-ammonium cation bridge two O1-anions over a centre of inversion to form a centrosymmetric, 12-membered {⋯HNH⋯OCO}_2_ synthon, Fig. 4 ▸ *b*. The N6-ammonium H atoms form similar bridges but with the O5-anion. The result is the formation of a three-dimensional architecture, Fig. 4 ▸ *c*.

## Hirshfeld surface analysis   

**Figure 5:**
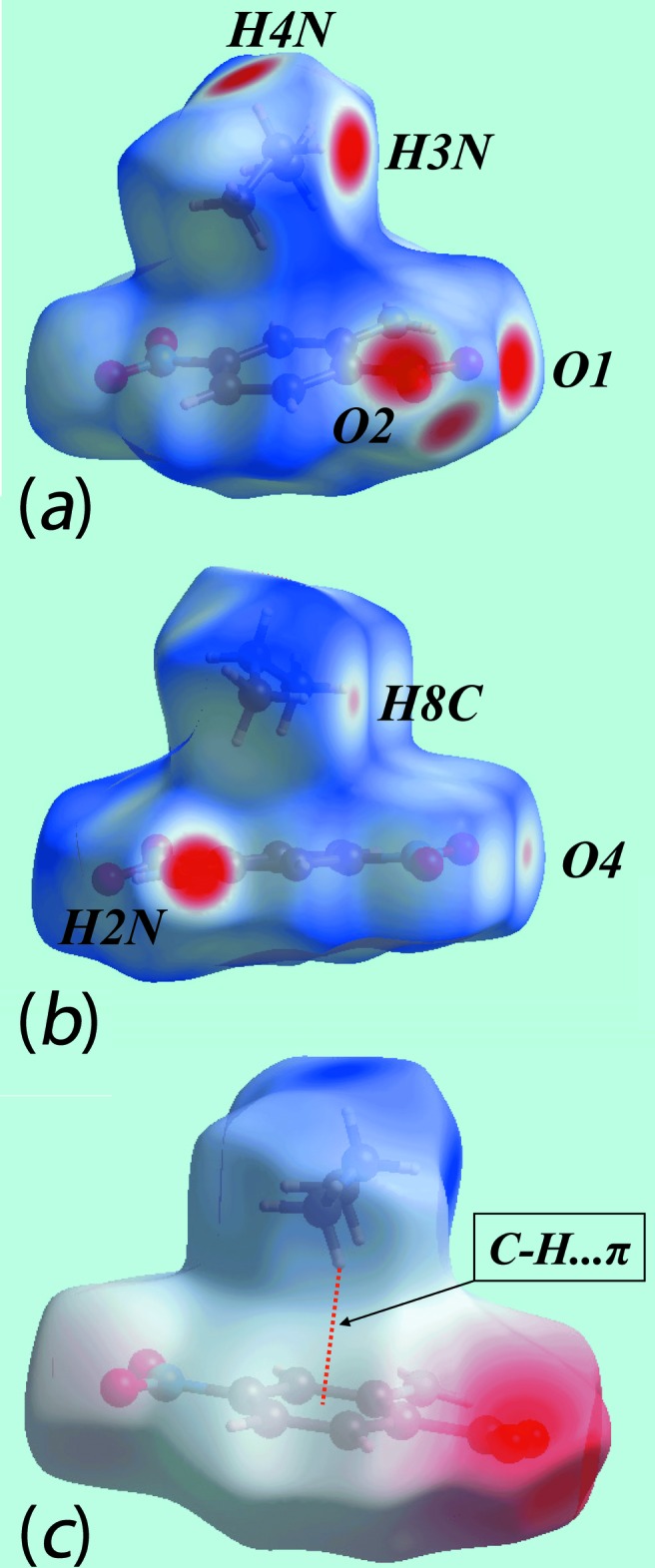
Views of Hirshfeld surfaces for (I) mapped over (*a*) and (*b*) *d*_norm_ and (*c*) the electrostatic potential (the red and blue regions represent negative and positive electrostatic potentials, respectively).

**Figure 6:**
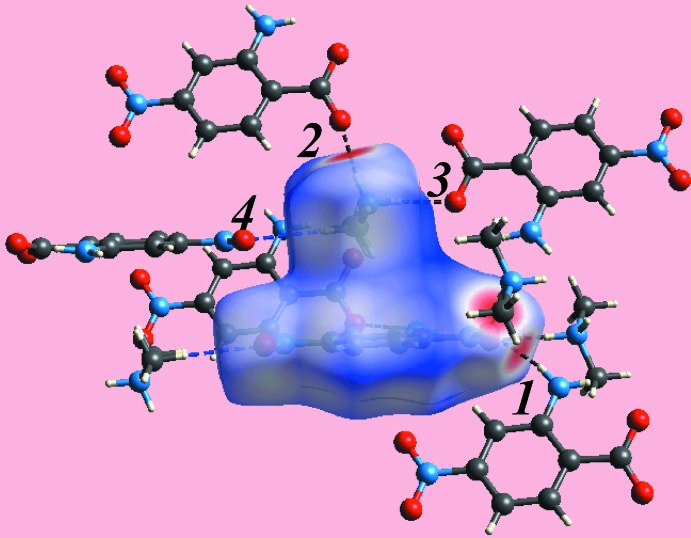
A view of Hirshfeld surface mapped over *d*_norm_ for (I), showing N—H⋯O hydrogen bonds about the reference mol­ecule. The hydrogen bonds are indicated with black dashed lines and are labelled as 1, 2 and 3. The inter­molecular C—H⋯O inter­action is indicated with a blue dashed line and with label 4.

Hirshfeld surface analysis for (I) and (II) was carried out as described previously (Cardoso *et al.*, 2016 ▸). In the two views of the Hirshfeld surface for (I) mapped over *d*_norm_ in the range −0.3 to + 1.8 au shown in Fig. 5 ▸ *a* and *b*, the bright-red spots appearing near the amino-H2*N*, ammonium-H3*N* and H4*N*, and carboxyl­ate-O1 and O2 atoms represent donors and acceptors of the dominating hydrogen bonds; they are viewed as blue and red regions on Hirshfeld surfaces mapped over electrostatic potential in the range −0.24 to + 0.31 au in Fig. 5 ▸ *c* and correspond to positive and negative potentials, respectively. The faint-red spots at the methyl-H8*C* and nitro-O4 atoms in Fig. 5 ▸ *b* are due to the presence of comparatively weak C—H⋯O inter­actions. Also from Fig. 5 ▸ *c*, it is evident that the electrostatic coulombic inter­action between the di­methyl­ammonium and 2-amino-4-nitro­benzoate species results in a cation–anion pair through a C—H⋯π contact between methyl-H9*C* and the benzene (C1–C6) ring, as highlighted by the dotted bond. The immediate environment about the ion-pair within the Hirshfeld surface mapped over *d*_norm_ mediated by the above inter­actions is illustrated in Fig. 6 ▸.

**Figure 7:**
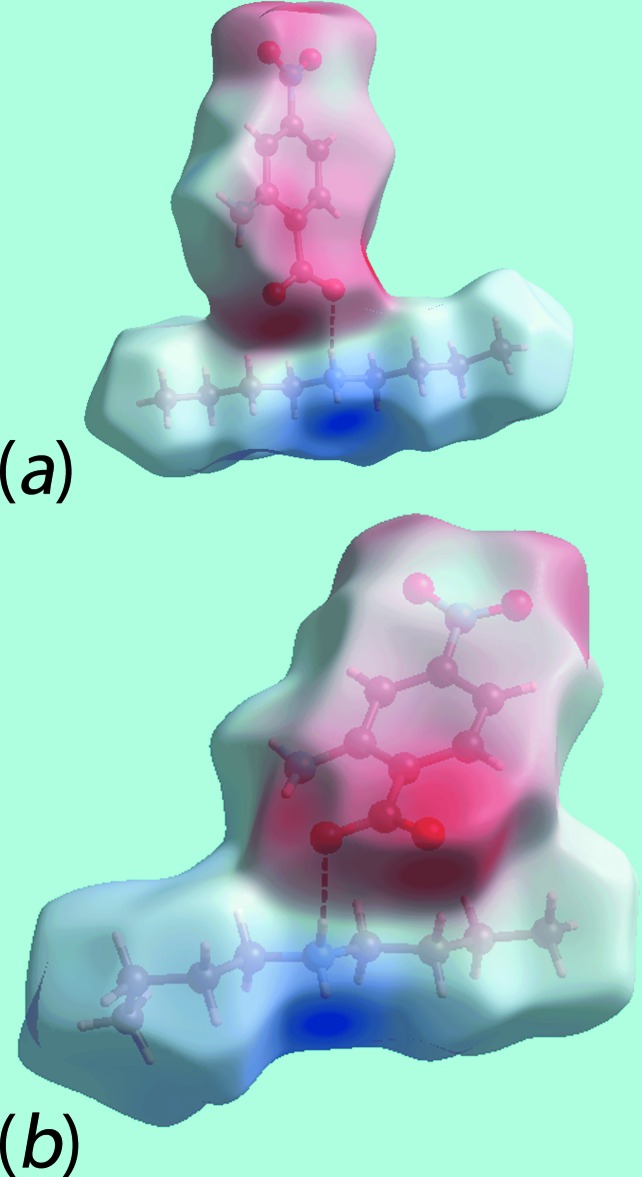
A view of Hirshfeld surface mapped over the electrostatic potential for (II) showing the N—H⋯O hydrogen bond leading to ion-pairs (*a*) 1 and (*b*) 2. The hydrogen bonds are indicated with black dashed lines.

In the crystal of the di­butyl­ammonium salt, (II), each of the two independent pairs of cations and anions are connected by charge-assisted ammonium-N—H⋯O(carboxyl­ate) hydrogen bonds. The Hirshfeld surfaces for each of the independent pairs, hereafter referred as ion-pair 1 (involving the N4-cation and O1-anion) and ion-pair 2 (involving the N3-cation and O5-anion), were generated as well that for the entire structure of (II). The Hirshfeld surfaces mapped over the electrostatic potential for the ion-pairs are shown in Fig. 7 ▸.

**Figure 8:**
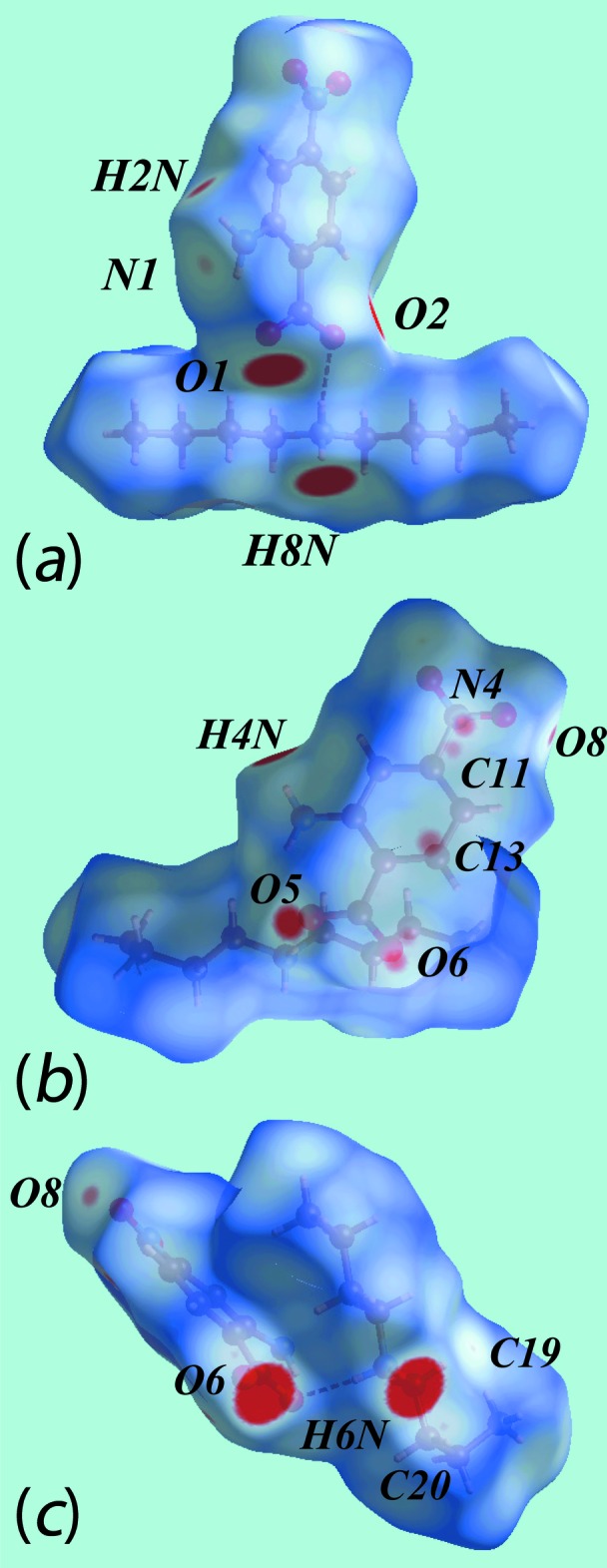
Views of Hirshfeld surfaces mapped over *d*_norm_ for (II), showing (*a*) ion-pair 1 in the range −0.2 to + 1.8 au, (*b*) ion-pair 2 in the range −0.2 to + 1.6 au and (*c*) ion-pair 2 in the range −0.1 to + 1.6 au.

**Figure 9:**
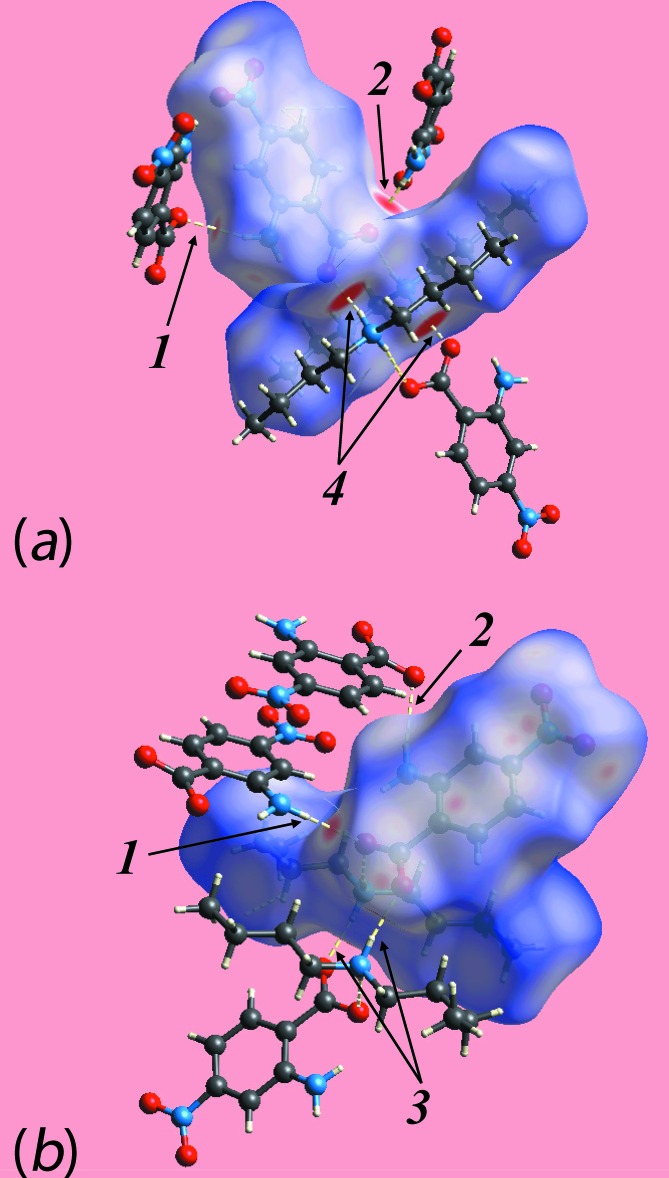
The immediate environment about reference ion-pairs within Hirshfeld surfaces mapped over *d*_norm_ showing N—H⋯O hydrogen bonding in (II), showing (*a*) ion-pair 1 and (*b*) ion-pair 2.

Views of Hirshfeld surfaces mapped over *d*_norm_, in the ranges −0.2 to +1.8 au for ion-pair 1, Fig. 8 ▸ *a*, −0.1 to +1.6 au for ion-pair 2, Fig. 8 ▸ *b*, and in order to reveal more detail (red-spots) on the surface, −0.1 to +1.6, for ion-pair 2, Fig. 8 ▸ *c*. The bright-red spots appearing near amino-H2*N* and H4*N*, ammonium-H6*N* and H8*N*, and carboxyl­ate-O1, O2, O5 and O6 atoms indicate donors and acceptors of charge-assisted N—H⋯O hydrogen bonds between the respective ion-pairs. The short inter­atomic O⋯H contact between the amino-H2*N* and nitro-O8 atoms, Table 3 ▸, is evident from the faint-red spots at the N1, Fig. 8 ▸ *a*, and nitro-O8 atoms, Fig. 8 ▸ *c*. The faint-red spots present in Fig. 8 ▸ *b* near atoms N4, C11, C13 and O6 of ion-pair 2 indicate their participation in short inter­atomic contacts in the crystal, Table 3 ▸. As the inter­molecular C—H⋯O inter­actions involving the butyl-C19- and C20-H atoms of ion-pair 2 are very weak compared to the above, they only appear as very faint spots in Fig. 8 ▸ *c*; the C27—H27*A*⋯O4 inter­action is even weaker than these, showing no spots even at the lower *d*_norm_ range. The immediate environments about the ion-pairs within *d*_norm_ mapped Hirshfeld surface mediated by N—H⋯O hydrogen-bonding inter­actions are illustrated in Fig. 9 ▸.

**Figure 10:**
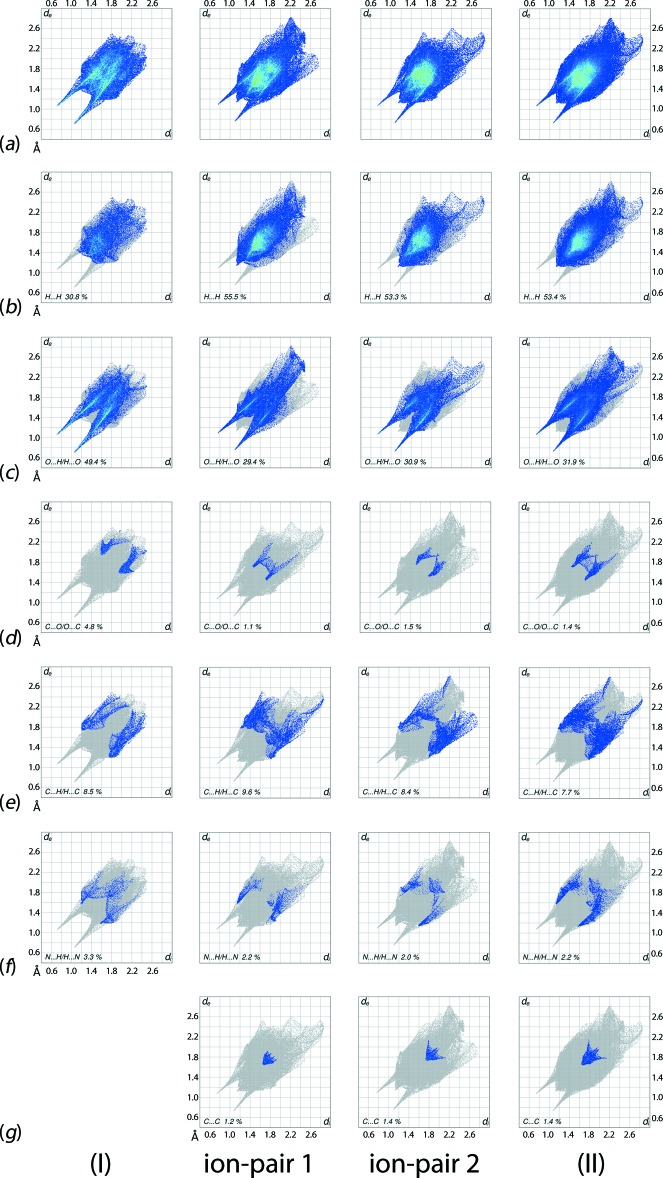
Comparison between (I), ion-pair 1 in (II), ion-pair 2 in (II) and (II) of the (*a*) full two-dimensional fingerprint plots, and the plots delineated into (*b*) H⋯H, (*c*) O⋯H/H⋯O, (*d*) C⋯O/O⋯C, (*e*) C⋯H/H⋯C, (*f*) N⋯H/H⋯N and (*g*) C⋯C contacts.

The overall two-dimensional fingerprint plots for (I), ion-pair 1 in (II), ion-pair 2 in (II) and (II), and those delineated into H⋯H, O⋯H/H⋯O, C⋯O/O⋯C, C⋯H/H⋯C, N⋯H/H⋯N and C⋯C contacts (McKinnon *et al.*, 2007 ▸) are shown in Fig. 10 ▸ *a*–*g*, respectively. The relative contributions from different contacts to the Hirshfeld surfaces of (I) and (II) are summarized in Table 4 ▸.

The fingerprint plot delineated into O⋯H/H⋯O contacts for (I), Fig. 10 ▸ *c*, shows that these contacts make the most significant contribution, *i.e*. almost half (49.4%), to the Hirshfeld surface. This may be due to salt formation through electrostatic inter­actions resulting in only a few hydrogen atoms being available on the surface to form inter­atomic H⋯H and other contacts. This is also reflected in a comparatively low contribution from H⋯H contacts to the Hirshfeld surface, Fig. 10 ▸ *b* and Table 4 ▸. A pair of long spikes with tips at *d*_e_ + *d*_i_ ∼1.8 Å in Fig. 10 ▸ *c* is the result of charge-assisted N—H⋯O hydrogen bonds, Table 1 ▸. The significant contributions from O⋯H/H⋯O to the Hirshfeld surfaces are also due to the presence of short inter­atomic O⋯H/H⋯O, C—H⋯O and N—H⋯O inter­actions, Tables 1 ▸ and 3 ▸. The fingerprint plot delineated into C⋯O/O⋯C contacts, Fig. 10 ▸ *d*, having a fin-like distribution of points with tips at *d*_e_ + *d*_i_ ∼3.5 Å and a 4.8% contribution to the surface, indicate the presence of influential N—O⋯π and C—H⋯O inter­actions in the crystal of (I), Tables 1 ▸ and 3 ▸. The 8.5% contribution from C⋯H/H.·C contacts, Fig. 10 ▸ *e*, is the result of a short inter­atomic contact, Table 3 ▸, and an intra-ion-pair methyl-C—H⋯π inter­action within the cation–anion pair.

In the structure of (II), the most significant contribution to the Hirshfeld surface is from H⋯H contacts, an observation clearly related to the hydrogen-rich *n*-butyl side chains in the cations, *cf*. (I). This is also reflected through the appearance of green points in the fingerprint plot delineated into H⋯H contacts, Fig. 10 ▸ *b*, and in the nearly same percentage contribution from these contacts in the plots for each ion-pair and overall Hirshfeld surface, Table 4 ▸. A pair of small peaks at *d*_e_ + *d*_i_ ∼2.2 Å in Fig. 10 ▸ *b* is the result of short inter­atomic H⋯H contacts in the crystal, Table 3 ▸.

A pair of long spikes with the tips at *d*_e_ + *d*_i_ ∼1.8 Å in the fingerprint delineated into O⋯H/H⋯O contacts, Fig. 10 ▸ *c*, are a result of the N—H⋯O hydrogen bonds. A pair of regions comprising aligned green points in the plot beginning at *d*_e_ + *d*_i_ ∼2.7 Å are due to short inter­atomic O⋯H/H⋯O contacts present in the structure, Table 3 ▸. The distinct shapes in the fingerprint plots delineated into C⋯H/H⋯C contacts for ion-pairs 1 and 2, Fig. 10 ▸ *e*, and their different percentage contributions to the respective Hirshfeld surfaces, Table 4 ▸, reflect the different conformations of the butyl chains in the cations; the small tips at *d*_e_ + *d*_i_ ∼2.9 Å in the overall plot indicate short inter­atomic C⋯H/H⋯C contacts, Table 3 ▸.

Though the inter­atomic N⋯H/H⋯N, C⋯O/O⋯C and C⋯C contacts each makes a small percentage contribution to the Hirshfeld surface of (II), they reflect recognizable inter­molecular inter­actions in the crystal. A short inter­atomic N⋯H/H⋯N contact between nitro-N2 and butyl-H21*B* is evident as a thin edge at *d*_e_ + *d*_i_ ∼2.7 Å in the overall fingerprint plot which results from the superposition of the individual plots for ion-pairs 1 and 2, Fig. 10 ▸ *f*. The overall 1.4% contribution from C⋯O/O⋯C contacts results from short inter­atomic C⋯O contacts, Table 3 ▸, and from C—H⋯O inter­actions involving butyl-C19, C20 and C27 and nitro-O4 and carboxyl­ate-O6 and O7 atoms, Table 3 ▸. These inter­molecular inter­actions are also viewed as a pair of short thick edges at *d*_e_ + *d*_i_ ∼3.2 Å in the overall fingerprint plot delineated into these contacts, Fig.10*d*.

The overall 1.4% contribution from C⋯C contacts to the Hirshfeld surfaces, Fig. 10 ▸ *g*, is the result of π–π stacking between inversion-related benzene (C1–C6) rings [*Cg*⋯*Cg* = 3.9250 (13) Å; symmetry operation: −*x*, −*y*, −*z*] of ion-pair 1 and a 1.2% contribution from short C⋯C contacts in ion-pair 2, Table 3 ▸. In the fingerprint plot, the presence of π–π stacking inter­action is viewed as a peak at *d*_e_ + *d*_i_ ∼3.4 Å, Fig. 10 ▸ *g*.

## Database survey   

As indicated in the *Chemical context*, a good number of ammonium salts of anions derived from 2-amino-4-nitro­benzoic acid have been described in the crystallographic literature. Salient geometric data for these are collated in Table 5 ▸. The consistent feature of the 2-amino-4-nitro­benzoate anions is deprotonation of the original carb­oxy­lic acid. Most of the dianions are relatively close to being planar with the outlier structures being the salts with [NH_4_]^+^ (Smith, 2014*b* ▸), with a dihedral angle of 26.4 (3)° between the C_6_ ring and the carboxyl­ate group, and (II) with a dihedral angle of 12.6 (3)° between the the nitro group and the C_6_ ring. The greatest twist between the carboxyl­ate and nitro substituents in any of the anions included in Table 3 ▸ is 24.1 (4)°, which also occurs in the aforementioned ammonium salt (Smith, 2014*b* ▸).

## Synthesis and crystallization   

The salts were isolated from the very similar reaction conditions. A solution of the respective *R*_2_NH amine (0.1 mmol) in EtOH (5 ml) and 4-nitro­anthranilic acid (0.1 mmol) in EtOH (10 ml) were mixed and left at room temperature. The yellow blocks of (I) and orange blocks of (II), which had formed after 4 days, were collected and used as such in the structure determinations. *R* = Me salt: M.p. 428–431 (*dec*.) K. IR (KBr, cm^−1^) 3400–2500 (*br*), 1630, 1553, 1424, 1347, 1267, 1078, 822, 724, 692, 573 cm^−1^. *R* = *n*-Bu salt: M.p. 415–417 (*dec*.) K. IR: 3400–2500 (*br*) 1626, 1535, 1535, 1348, 1323, 1261, 825, 735 cm^−1^.

## Refinement   

Crystal data, data collection and structure refinement details are summarized in Table 6 ▸. Carbon-bound H atoms were placed in calculated positions (C—H = 0.95–0.99 Å) and were included in the refinement in the riding-model approximation, with *U*_iso_(H) set to 1.2–1.5*U*_eq_(C). The N-bound H atoms were located from difference maps but, refined with N—H = 0.88±0.01 Å, and with *U*_iso_(H) = 1.2*U*_eq_(N). In (II), owing to poor agreement, one reflection, *i.e*. (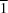 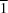
1), was omitted. Further, the maximum and minimum residual electron density peaks of 0.85 and 0.40 e Å^−3^, respectively, were located 0.92 and 0.64 Å from the H21*A* and C22 atoms, respectively.

## Supplementary Material

Crystal structure: contains datablock(s) I, II, global. DOI: 10.1107/S2056989016017266/hb7627sup1.cif

Structure factors: contains datablock(s) I. DOI: 10.1107/S2056989016017266/hb7627Isup2.hkl

Structure factors: contains datablock(s) II. DOI: 10.1107/S2056989016017266/hb7627IIsup3.hkl

Supporting information file. DOI: 10.1107/S2056989016017266/hb7627Isup4.cml

Supporting information file. DOI: 10.1107/S2056989016017266/hb7627IIsup5.cml

CCDC references: 1511822, 1511821

Additional supporting information:  crystallographic information; 3D view; checkCIF report

## Figures and Tables

**Table 1:** Hydrogen-bond geometry (Å, °) for (I) *Cg*1 is the centroid of the (C1–C6) ring.

*D*—H⋯*A*	*D*—H	H⋯*A*	*D*⋯*A*	*D*—H⋯*A*
N1—H1*N*⋯O1	0.869 (18)	2.012 (18)	2.6694 (17)	131.6 (15)
N1—H2*N*⋯O2^i^	0.889 (18)	2.019 (18)	2.8900 (16)	166.2 (16)
N3—H3*N*⋯O1^ii^	0.944 (17)	1.774 (17)	2.7141 (15)	173.4 (15)
N3—H4*N*⋯O2^iii^	0.919 (16)	1.834 (15)	2.7385 (15)	167.6 (15)
C8—H8*C*⋯O4^iv^	0.98	2.48	3.4589 (19)	174
N2—O4⋯*Cg*1^v^	1.23 (1)	3.40 (1)	4.3668 (14)	136 (1)
C9—H9*C*⋯*Cg*1	0.98	2.64	3.5512 (16)	154

Symmetry codes: (i) 
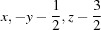
; (ii) 
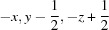
; (iii) 
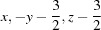
; (iv) 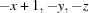; (v) 
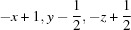
.

**Table 2:** Hydrogen-bond geometry (Å, °) for (II)

*D*—H⋯*A*	*D*—H	H⋯*A*	*D*⋯*A*	*D*—H⋯*A*
N1—H1*N*⋯O1	0.88 (1)	1.98 (2)	2.696 (2)	137 (2)
N1—H2*N*⋯O5^i^	0.88 (2)	2.38 (2)	3.226 (3)	160 (2)
N3—H3*N*⋯O5	0.88 (2)	2.01 (2)	2.714 (3)	136 (2)
N3—H4*N*⋯O2^ii^	0.88 (2)	2.19 (2)	3.052 (2)	168 (2)
N5—H5*N*⋯O5	0.88 (2)	1.89 (2)	2.757 (3)	166 (2)
N5—H6*N*⋯O6^iii^	0.89 (2)	1.81 (2)	2.697 (3)	173 (2)
N6—H7*N*⋯O2	0.89 (2)	1.89 (2)	2.759 (2)	167 (2)
N6—H8*N*⋯O1^iv^	0.89 (2)	1.85 (2)	2.712 (2)	163 (2)
C19—H19*A*⋯O7^v^	0.99	2.56	3.343 (3)	136
C20—H20*B*⋯O6^iii^	0.99	2.56	3.297 (3)	131
C27—H27*A*⋯O4^vi^	0.99	2.57	3.550 (3)	169

Symmetry codes: (i) 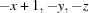; (ii) 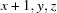; (iii) 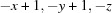; (iv) 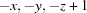; (v) 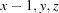; (vi) 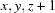.

**Table 3:** Summary of short inter­atomic contacts (Å) in (I) and (II)

Contact	Distance	Symmetry operation
(I)		
O4⋯H9*B*	2.70	1 − *x*, 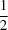 + *y*, 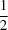 − *z*
C3⋯H8*A*	2.89	*x*, *y*, *z*
(II)		
O8⋯H2*N*	2.70 (2)	1 + *x*, 1 + *y*, *z*
O6⋯N4	2.994 (2)	2 − *x*, 1 − *y*, −*z*
O6⋯C11	3.179 (3)	2 − *x*, 1 − *y*, −*z*
C13⋯C13	3.310 (3)	2 − *x*, 1 − *y*, −*z*
H19*A*.·O7	2.56	−1 + *x*, *y*, *z*
H20*B*⋯O6	2.56	1 − *x*, 1 − *y*, −*z*
H27*A*⋯O4	2.57	*x*, *y*, 1 + *z*
H3⋯H3*N*	2.26	1 − *x*, 1 − *y*, −*z*
H5⋯H13	2.33	1 − *x*, 1 − *y*, −*z*
H18*B*⋯H22*B*	2.37	*x*, 1 + *y*, *z*
O1⋯H28*A*	2.66	*x*, *y*, *z*
O5⋯H3	2.70	1 − *x*, −*y*, *z*
O7⋯H25*A*	2.64	1 − *x*, 1 − *y*, 1 − *z*
O8⋯H25*A*	2.66	1 − *x*, 1 − *y*, 1 − *z*
C7⋯H4*N*	2.89 (2)	−1 + *x*, *y*, *z*
C7⋯H7*N*	2.76 (2)	*x*, *y*, *z*
C7⋯H8*N*	2.78 (2)	−*x*, −*y*, 1 − *z*
C10⋯H6	2.89	1 + *x*, *y*, *z*
C14⋯H6*N*	2.78 (2)	1 − *x*, 1 − *y*, −*z*
C22⋯H27*B*	2.83	*x*, *y*, *z*
N2⋯H21*B*	2.72	1 − *x*, −*y*, −*z*
C12⋯C14	3.391 (3)	2 − *x*, 1 − *y*, −*z*

**Table 4:** Percentage contribution to inter­atomic contacts from the Hirshfeld surface for (I) and (II)

Contact	(I)	(II) - pair 1	(II) - pair 2	(II)
H⋯H	30.8	55.5	53.3	53.4
O⋯H/H⋯O	49.4	29.4	30.9	31.9
C⋯H/H⋯C	8.5	8.4	9.6	7.7
C⋯O/O⋯C	4.8	1.5	1.1	1.4
N⋯H/H⋯N	3.3	2.0	2.2	2.2
O⋯O	2.3	0.3	0.7	0.6
N⋯O/O⋯N	0.9	0.3	1.0	0.7
C⋯ C	0.0	1.4	1.2	1.4
C⋯N/N⋯C	0.0	0.7	0.0	0.4
N⋯ N	0.0	0.5	0.0	0.3

**Table 5:** Geometric data (°) for ammonium salts of 2-amino-4-nitro­benzoate

cation	*Z*′	C_6_/CO_2_	C_6_/NO_2_	CO_2_/NO_2_	Ref.
[NH_4_]^+^	1	26.4 (3)	2.9 (3)	24.1 (4)	Smith (2014*b* ▸)
[Cy_2_NH_2_]^+^	2	9.87 (10)	7.58 (15)	3.42 (19)	Smith *et al.* (2004 ▸)
		9.52 (9)	7.86 (11)	3.92 (2)	
[(H_2_N)_2_C=NH_2_]^+^	1	5.88 (11)	5.64 (12)		Smith *et al.* (2007 ▸)
[O(CH_2_CH_2_)_2_NH_2_]^+^	1	17.92 (9)	1.28 (11)	19.19 (13)	Smith & Lynch (2016 ▸)
[H_3_NCH_2_CH_2_NH_3_]^2+^	1	3.44 (14)	0.69 (11)	3.2 (2)	Smith *et al.* (2002 ▸)
[Me_2_NH_2_]^+^	1	11.45 (13)	3.71 (15)	7.9 (2)	this work
[*n*-Bu_2_NH_2_]^+^	2	12.73 (6)	4.30 (10)	17.02 (8)	this work
		8.1 (4)	12.6 (3)	19.0 (5)	

**Table 6:** Experimental details

	(I)	(II)
Crystal data		
Chemical formula	C_2_H_8_N^+^·C_7_H_5_N_2_O_4_^−^	C_8_H_20_N^+^·C_7_H_5_N_2_O_4_^−^
*M*_r_	227.22	311.38
Crystal system, space group	Monoclinic, *P*2_1_/*c*	Triclinic, *P* 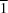
Temperature (K)	120	120
*a*, *b*, *c* (Å)	11.2593 (5), 7.5563 (2), 13.0437 (6)	11.1615 (3), 12.5172 (4), 13.2399 (4)
α, β, γ (°)	90, 96.716 (2), 90	82.405 (1), 78.107 (2), 70.915 (2)
*V* (Å^3^)	1102.13 (8)	1706.36 (9)
*Z*	4	4
Radiation type	Mo *K*α	Mo *K*α
μ (mm^−1^)	0.11	0.09
Crystal size (mm)	0.35 × 0.25 × 0.16	0.20 × 0.14 × 0.12

Data collection		
Diffractometer	Bruker–Nonius Roper CCD camera on κ-goniostat	Bruker–Nonius Roper CCD camera on κ-goniostat
Absorption correction	Multi-scan (*SADABS*; Sheldrick, 2007 ▸)	Multi-scan (*SADABS*; Sheldrick, 2007 ▸)
*T*_min_, *T*_max_	0.649, 0.746	0.665, 0.746
No. of measured, independent and observed [*I* > 2σ(*I*)] reflections	15431, 2537, 1952	34976, 7811, 5022
*R*_int_	0.051	0.074
(sin θ/λ)_max_ (Å^−1^)	0.650	0.650

Refinement		
*R*[*F* ^2^ > 2σ(*F* ^2^)], *wR*(*F* ^2^), *S*	0.043, 0.125, 1.01	0.062, 0.180, 1.03
No. of reflections	2537	7811
No. of parameters	159	425
No. of restraints	0	8
Δρ_max_, Δρ_min_ (e Å^−3^)	0.24, −0.31	0.85, −0.40

Computer programs: *DENZO* (Otwinowski & Minor, 1997 ▸), *COLLECT* (Hooft, 1998 ▸), *SHELXS97* (Sheldrick, 2008 ▸), *SHELXL2014* (Sheldrick, 2015 ▸), *ORTEP-3 for Windows* (Farrugia, 2012 ▸), *DIAMOND* (Brandenburg, 2006 ▸) and *publCIF* (Westrip, 2010 ▸).

## supplementary crystallographic information

### (I) Dimethylazanium 2-amino-4-nitrobenzoate Crystal data

**Table:**

C_2_H_8_N^+^·C_7_H_5_N_2_O_4_^−^	*F*(000) = 480
*M**_r_* = 227.22	*D*_x_ = 1.369 Mg m^−^^3^
Monoclinic, *P*2_1_/*c*	Mo *K*α radiation, λ = 0.71073 Å
*a* = 11.2593 (5) Å	Cell parameters from 11561 reflections
*b* = 7.5563 (2) Å	θ = 2.9–27.5°
*c* = 13.0437 (6) Å	µ = 0.11 mm^−^^1^
β = 96.716 (2)°	*T* = 120 K
*V* = 1102.13 (8) Å^3^	Block, yellow
*Z* = 4	0.35 × 0.25 × 0.16 mm

### (I) Dimethylazanium 2-amino-4-nitrobenzoate Data collection

**Table:**

Bruker–Nonius Roper CCD camera on κ-goniostat diffractometer	2537 independent reflections
Radiation source: Bruker–Nonius FR591 rotating anode	1952 reflections with *I* > 2σ(*I*)
Graphite monochromator	*R*_int_ = 0.051
Detector resolution: 9.091 pixels mm^-1^	θ_max_ = 27.5°, θ_min_ = 3.1°
φ & ω scans	*h* = −14→14
Absorption correction: multi-scan (SADABS; Sheldrick, 2007)	*k* = −9→9
*T*_min_ = 0.649, *T*_max_ = 0.746	*l* = −16→16
15431 measured reflections	

### (I) Dimethylazanium 2-amino-4-nitrobenzoate Refinement

**Table:**

Refinement on *F*^2^	0 restraints
Least-squares matrix: full	Hydrogen site location: mixed
*R*[*F*^2^ > 2σ(*F*^2^)] = 0.043	*w* = 1/[σ^2^(*F*_o_^2^) + (0.0704*P*)^2^ + 0.2595*P*] where *P* = (*F*_o_^2^ + 2*F*_c_^2^)/3
*wR*(*F*^2^) = 0.125	(Δ/σ)_max_ < 0.001
*S* = 1.01	Δρ_max_ = 0.24 e Å^−^^3^
2537 reflections	Δρ_min_ = −0.31 e Å^−^^3^
159 parameters	

### (I) Dimethylazanium 2-amino-4-nitrobenzoate Special details

**Table:**

Geometry. All esds (except the esd in the dihedral angle between two l.s. planes) are estimated using the full covariance matrix. The cell esds are taken into account individually in the estimation of esds in distances, angles and torsion angles; correlations between esds in cell parameters are only used when they are defined by crystal symmetry. An approximate (isotropic) treatment of cell esds is used for estimating esds involving l.s. planes.

### (I) Dimethylazanium 2-amino-4-nitrobenzoate Fractional atomic coordinates and isotropic or equivalent isotropic displacement parameters (Å^2^)

**Table:**

	*x*	*y*	*z*	*U*_iso_*/*U*_eq_	
O1	0.03429 (9)	0.26005 (14)	0.33705 (8)	0.0267 (3)	
O2	0.13527 (9)	0.07211 (13)	0.44549 (7)	0.0247 (3)	
O3	0.51006 (10)	0.21618 (17)	0.03910 (9)	0.0389 (3)	
O4	0.61013 (10)	0.06520 (18)	0.16030 (9)	0.0421 (3)	
N1	0.11153 (11)	0.31535 (17)	0.15387 (10)	0.0251 (3)	
H1N	0.0543 (16)	0.334 (2)	0.1919 (14)	0.030*	
H2N	0.1060 (15)	0.355 (2)	0.0893 (14)	0.030*	
N2	0.51972 (10)	0.14316 (17)	0.12332 (9)	0.0260 (3)	
C1	0.22560 (12)	0.16283 (17)	0.29838 (10)	0.0179 (3)	
C2	0.21430 (12)	0.23829 (17)	0.19798 (10)	0.0184 (3)	
C3	0.31350 (12)	0.22848 (18)	0.14090 (10)	0.0197 (3)	
H3	0.3084	0.2758	0.0730	0.024*	
C4	0.41748 (12)	0.14978 (18)	0.18468 (11)	0.0210 (3)	
C5	0.43157 (12)	0.07721 (18)	0.28298 (11)	0.0219 (3)	
H5	0.5049	0.0248	0.3113	0.026*	
C6	0.33365 (12)	0.08477 (18)	0.33791 (11)	0.0214 (3)	
H6	0.3403	0.0348	0.4052	0.026*	
C7	0.12404 (12)	0.16464 (17)	0.36448 (10)	0.0193 (3)	
N3	0.14643 (10)	−0.25004 (16)	0.04241 (9)	0.0220 (3)	
H3N	0.0796 (15)	−0.249 (2)	0.0799 (13)	0.026*	
H4N	0.1435 (14)	−0.349 (2)	0.0013 (13)	0.026*	
C8	0.14422 (13)	−0.0913 (2)	−0.02465 (12)	0.0270 (3)	
H8A	0.1384	0.0153	0.0173	0.041*	
H8B	0.0750	−0.0976	−0.0776	0.041*	
H8C	0.2178	−0.0866	−0.0579	0.041*	
C9	0.25270 (13)	−0.2585 (2)	0.12109 (12)	0.0281 (3)	
H9A	0.3256	−0.2555	0.0867	0.042*	
H9B	0.2506	−0.3686	0.1606	0.042*	
H9C	0.2521	−0.1571	0.1679	0.042*	

### (I) Dimethylazanium 2-amino-4-nitrobenzoate Atomic displacement parameters (Å^2^)

**Table:**

	*U*^11^	*U*^22^	*U*^33^	*U*^12^	*U*^13^	*U*^23^
O1	0.0227 (5)	0.0347 (6)	0.0244 (5)	0.0062 (4)	0.0091 (4)	0.0055 (4)
O2	0.0312 (6)	0.0269 (5)	0.0173 (5)	−0.0004 (4)	0.0084 (4)	0.0029 (4)
O3	0.0322 (6)	0.0597 (8)	0.0275 (6)	0.0024 (5)	0.0153 (5)	0.0093 (5)
O4	0.0229 (6)	0.0656 (8)	0.0395 (7)	0.0111 (5)	0.0106 (5)	0.0060 (6)
N1	0.0211 (6)	0.0369 (7)	0.0182 (6)	0.0052 (5)	0.0062 (5)	0.0073 (5)
N2	0.0206 (6)	0.0340 (7)	0.0245 (7)	−0.0022 (5)	0.0072 (5)	−0.0024 (5)
C1	0.0199 (7)	0.0183 (7)	0.0162 (7)	−0.0022 (5)	0.0049 (5)	−0.0016 (5)
C2	0.0201 (7)	0.0187 (6)	0.0165 (7)	−0.0029 (5)	0.0027 (5)	−0.0015 (5)
C3	0.0218 (7)	0.0228 (7)	0.0149 (6)	−0.0028 (5)	0.0042 (5)	0.0003 (5)
C4	0.0189 (7)	0.0244 (7)	0.0209 (7)	−0.0034 (5)	0.0075 (5)	−0.0041 (5)
C5	0.0192 (7)	0.0248 (7)	0.0216 (7)	0.0008 (5)	0.0016 (5)	−0.0005 (5)
C6	0.0245 (7)	0.0223 (7)	0.0174 (7)	−0.0010 (5)	0.0023 (5)	0.0014 (5)
C7	0.0219 (7)	0.0207 (7)	0.0157 (7)	−0.0035 (5)	0.0042 (5)	−0.0021 (5)
N3	0.0202 (6)	0.0245 (6)	0.0223 (6)	−0.0031 (5)	0.0067 (5)	−0.0031 (5)
C8	0.0256 (8)	0.0287 (8)	0.0275 (8)	−0.0006 (6)	0.0063 (6)	0.0024 (6)
C9	0.0242 (8)	0.0299 (8)	0.0301 (8)	−0.0026 (6)	0.0024 (6)	−0.0009 (6)

### (I) Dimethylazanium 2-amino-4-nitrobenzoate Geometric parameters (Å, º)

**Table:**

O1—C7	1.2587 (17)	C4—C5	1.387 (2)
O2—C7	1.2609 (16)	C5—C6	1.3845 (19)
O3—N2	1.2227 (16)	C5—H5	0.9500
O4—N2	1.2252 (16)	C6—H6	0.9500
N1—C2	1.3614 (18)	N3—C8	1.4833 (19)
N1—H1N	0.870 (18)	N3—C9	1.4838 (19)
N1—H2N	0.890 (18)	N3—H3N	0.944 (18)
N2—C4	1.4777 (17)	N3—H4N	0.917 (17)
C1—C6	1.3953 (19)	C8—H8A	0.9800
C1—C2	1.4203 (19)	C8—H8B	0.9800
C1—C7	1.5106 (18)	C8—H8C	0.9800
C2—C3	1.4148 (19)	C9—H9A	0.9800
C3—C4	1.376 (2)	C9—H9B	0.9800
C3—H3	0.9500	C9—H9C	0.9800
			
C2—N1—H1N	118.6 (12)	C1—C6—H6	118.7
C2—N1—H2N	120.5 (11)	O1—C7—O2	123.67 (12)
H1N—N1—H2N	120.6 (16)	O1—C7—C1	118.62 (12)
O3—N2—O4	123.59 (12)	O2—C7—C1	117.70 (12)
O3—N2—C4	118.57 (12)	C8—N3—C9	113.54 (11)
O4—N2—C4	117.84 (12)	C8—N3—H3N	109.9 (10)
C6—C1—C2	119.38 (12)	C9—N3—H3N	105.6 (10)
C6—C1—C7	118.58 (12)	C8—N3—H4N	108.4 (10)
C2—C1—C7	122.04 (12)	C9—N3—H4N	109.9 (10)
N1—C2—C3	118.96 (12)	H3N—N3—H4N	109.5 (14)
N1—C2—C1	122.78 (12)	N3—C8—H8A	109.5
C3—C2—C1	118.23 (12)	N3—C8—H8B	109.5
C4—C3—C2	119.33 (12)	H8A—C8—H8B	109.5
C4—C3—H3	120.3	N3—C8—H8C	109.5
C2—C3—H3	120.3	H8A—C8—H8C	109.5
C3—C4—C5	123.67 (12)	H8B—C8—H8C	109.5
C3—C4—N2	117.95 (12)	N3—C9—H9A	109.5
C5—C4—N2	118.38 (12)	N3—C9—H9B	109.5
C6—C5—C4	116.76 (13)	H9A—C9—H9B	109.5
C6—C5—H5	121.6	N3—C9—H9C	109.5
C4—C5—H5	121.6	H9A—C9—H9C	109.5
C5—C6—C1	122.61 (13)	H9B—C9—H9C	109.5
C5—C6—H6	118.7		
			
C6—C1—C2—N1	179.42 (13)	O4—N2—C4—C5	3.84 (19)
C7—C1—C2—N1	−0.8 (2)	C3—C4—C5—C6	0.7 (2)
C6—C1—C2—C3	1.04 (19)	N2—C4—C5—C6	−179.58 (12)
C7—C1—C2—C3	−179.14 (11)	C4—C5—C6—C1	−0.8 (2)
N1—C2—C3—C4	−179.59 (13)	C2—C1—C6—C5	−0.1 (2)
C1—C2—C3—C4	−1.14 (19)	C7—C1—C6—C5	−179.91 (12)
C2—C3—C4—C5	0.3 (2)	C6—C1—C7—O1	168.10 (13)
C2—C3—C4—N2	−179.47 (11)	C2—C1—C7—O1	−11.72 (19)
O3—N2—C4—C3	4.04 (19)	C6—C1—C7—O2	−10.67 (18)
O4—N2—C4—C3	−176.38 (13)	C2—C1—C7—O2	169.51 (12)
O3—N2—C4—C5	−175.73 (13)		

### (I) Dimethylazanium 2-amino-4-nitrobenzoate Hydrogen-bond geometry (Å, º)

Cg1 is the centroid of the (C1–C6) ring.

**Table:**

*D*—H···*A*	*D*—H	H···*A*	*D*···*A*	*D*—H···*A*
N1—H1*N*···O1	0.869 (18)	2.012 (18)	2.6694 (17)	131.6 (15)
N1—H2*N*···O2^i^	0.889 (18)	2.019 (18)	2.8900 (16)	166.2 (16)
N3—H3*N*···O1^ii^	0.944 (17)	1.774 (17)	2.7141 (15)	173.4 (15)
N3—H4*N*···O2^iii^	0.919 (16)	1.834 (15)	2.7385 (15)	167.6 (15)
C8—H8*C*···O4^iv^	0.98	2.48	3.4589 (19)	174
N2—O4···*Cg*1^v^	1.23 (1)	3.40 (1)	4.3668 (14)	136 (1)
C9—H9*C*···*Cg*1	0.98	2.64	3.5512 (16)	154

Symmetry codes: (i) *x*, −*y*−1/2, *z*−3/2; (ii) −*x*, *y*−1/2, −*z*+1/2; (iii) *x*, −*y*−3/2, *z*−3/2; (iv) −*x*+1, −*y*, −*z*; (v) −*x*+1, *y*−1/2, −*z*+1/2.

### (II) Dibutylazanium 2-amino-4-nitrobenzoate Crystal data

**Table:**

C_8_H_20_N^+^·C_7_H_5_N_2_O_4_^−^	*Z* = 4
*M**_r_* = 311.38	*F*(000) = 672
Triclinic, *P*1	*D*_x_ = 1.212 Mg m^−^^3^
*a* = 11.1615 (3) Å	Mo *K*α radiation, λ = 0.71073 Å
*b* = 12.5172 (4) Å	Cell parameters from 14576 reflections
*c* = 13.2399 (4) Å	θ = 2.9–27.5°
α = 82.405 (1)°	µ = 0.09 mm^−^^1^
β = 78.107 (2)°	*T* = 120 K
γ = 70.915 (2)°	Block, orange
*V* = 1706.36 (9) Å^3^	0.20 × 0.14 × 0.12 mm

### (II) Dibutylazanium 2-amino-4-nitrobenzoate Data collection

**Table:**

Bruker–Nonius Roper CCD camera on κ-goniostat diffractometer	7811 independent reflections
Radiation source: Bruker–Nonius FR591 rotating anode	5022 reflections with *I* > 2σ(*I*)
Graphite monochromator	*R*_int_ = 0.074
Detector resolution: 9.091 pixels mm^-1^	θ_max_ = 27.5°, θ_min_ = 3.0°
φ & ω scans	*h* = −14→14
Absorption correction: multi-scan (SADABS; Sheldrick, 2007)	*k* = −16→16
*T*_min_ = 0.665, *T*_max_ = 0.746	*l* = −17→17
34976 measured reflections	

### (II) Dibutylazanium 2-amino-4-nitrobenzoate Refinement

**Table:**

Refinement on *F*^2^	8 restraints
Least-squares matrix: full	Hydrogen site location: mixed
*R*[*F*^2^ > 2σ(*F*^2^)] = 0.062	*w* = 1/[σ^2^(*F*_o_^2^) + (0.0907*P*)^2^ + 0.4569*P*] where *P* = (*F*_o_^2^ + 2*F*_c_^2^)/3
*wR*(*F*^2^) = 0.180	(Δ/σ)_max_ < 0.001
*S* = 1.03	Δρ_max_ = 0.85 e Å^−^^3^
7811 reflections	Δρ_min_ = −0.40 e Å^−^^3^
425 parameters	

### (II) Dibutylazanium 2-amino-4-nitrobenzoate Special details

**Table:**

Geometry. All esds (except the esd in the dihedral angle between two l.s. planes) are estimated using the full covariance matrix. The cell esds are taken into account individually in the estimation of esds in distances, angles and torsion angles; correlations between esds in cell parameters are only used when they are defined by crystal symmetry. An approximate (isotropic) treatment of cell esds is used for estimating esds involving l.s. planes.

### (II) Dibutylazanium 2-amino-4-nitrobenzoate Fractional atomic coordinates and isotropic or equivalent isotropic displacement parameters (Å^2^)

**Table:**

	*x*	*y*	*z*	*U*_iso_*/*U*_eq_	
O1	0.09438 (14)	−0.08417 (13)	0.31913 (11)	0.0291 (4)	
O2	0.04512 (14)	0.10463 (12)	0.30797 (11)	0.0269 (3)	
O3	0.20429 (17)	−0.04415 (15)	−0.22162 (12)	0.0406 (4)	
O4	0.21251 (19)	0.12584 (15)	−0.22089 (13)	0.0457 (5)	
N1	0.1320 (2)	−0.18640 (17)	0.14324 (15)	0.0346 (5)	
H1N	0.133 (2)	−0.191 (2)	0.2101 (9)	0.042*	
H2N	0.161 (2)	−0.2458 (15)	0.1058 (18)	0.042*	
N2	0.19793 (18)	0.03761 (17)	−0.17638 (14)	0.0307 (4)	
C1	0.12087 (19)	0.01094 (17)	0.15175 (15)	0.0223 (4)	
C2	0.14160 (19)	−0.08524 (17)	0.09664 (16)	0.0231 (4)	
C3	0.1667 (2)	−0.07341 (18)	−0.01259 (16)	0.0265 (5)	
H3	0.1786	−0.1356	−0.0517	0.032*	
C4	0.1740 (2)	0.02861 (18)	−0.06210 (15)	0.0250 (5)	
C5	0.1602 (2)	0.12205 (18)	−0.01090 (16)	0.0275 (5)	
H5	0.1692	0.1904	−0.0473	0.033*	
C6	0.1326 (2)	0.11159 (18)	0.09631 (16)	0.0256 (5)	
H6	0.1212	0.1750	0.1336	0.031*	
C7	0.08429 (19)	0.00996 (18)	0.26807 (16)	0.0236 (4)	
O5	0.72446 (14)	0.36339 (13)	0.03271 (12)	0.0295 (4)	
O6	0.72498 (15)	0.53352 (13)	−0.04335 (12)	0.0317 (4)	
O7	1.17970 (15)	0.40957 (13)	0.29913 (12)	0.0338 (4)	
O8	1.13244 (16)	0.58920 (14)	0.25348 (13)	0.0364 (4)	
N3	0.84740 (18)	0.28457 (16)	0.19704 (14)	0.0273 (4)	
H3N	0.803 (2)	0.274 (2)	0.1533 (15)	0.033*	
H4N	0.8967 (19)	0.2265 (14)	0.2297 (17)	0.033*	
N4	1.11558 (18)	0.49651 (16)	0.25530 (14)	0.0276 (4)	
C8	0.83927 (19)	0.46491 (17)	0.09567 (15)	0.0230 (4)	
C9	0.88906 (19)	0.37761 (17)	0.17018 (16)	0.0228 (4)	
C10	0.98276 (19)	0.39011 (17)	0.22045 (15)	0.0225 (4)	
H10	1.0238	0.3304	0.2660	0.027*	
C11	1.0146 (2)	0.48871 (18)	0.20342 (15)	0.0239 (4)	
C12	0.9579 (2)	0.58021 (18)	0.13950 (17)	0.0273 (5)	
H12	0.9762	0.6499	0.1339	0.033*	
C13	0.8730 (2)	0.56440 (18)	0.08413 (17)	0.0271 (5)	
H13	0.8362	0.6237	0.0364	0.033*	
C14	0.75672 (19)	0.45293 (18)	0.02314 (16)	0.0250 (5)	
N5	0.47820 (19)	0.39824 (17)	0.14284 (15)	0.0326 (5)	
H5N	0.5508 (15)	0.393 (2)	0.0988 (16)	0.039*	
H6N	0.4105 (17)	0.427 (2)	0.1109 (18)	0.039*	
C15	0.4777 (2)	0.4736 (2)	0.22127 (19)	0.0390 (6)	
H15A	0.5582	0.4420	0.2503	0.047*	
H15B	0.4046	0.4754	0.2786	0.047*	
C16	0.4667 (2)	0.5925 (2)	0.17610 (19)	0.0383 (6)	
H16A	0.3855	0.6248	0.1482	0.046*	
H16B	0.5391	0.5908	0.1181	0.046*	
C17	0.4685 (4)	0.6673 (3)	0.2558 (3)	0.0668 (9)	
H17A	0.3895	0.6770	0.3087	0.080*	
H17B	0.5432	0.6287	0.2909	0.080*	
C18	0.4764 (4)	0.7816 (3)	0.2122 (3)	0.0786 (11)	
H18A	0.5545	0.7730	0.1599	0.118*	
H18B	0.4791	0.8248	0.2678	0.118*	
H18C	0.4007	0.8220	0.1802	0.118*	
C19	0.4816 (2)	0.2812 (2)	0.1868 (2)	0.0381 (6)	
H19A	0.4137	0.2859	0.2488	0.046*	
H19B	0.5658	0.2421	0.2086	0.046*	
C20	0.4617 (3)	0.2132 (2)	0.1099 (2)	0.0419 (6)	
H20A	0.5314	0.2068	0.0490	0.050*	
H20B	0.3791	0.2544	0.0861	0.050*	
C21	0.4596 (3)	0.0938 (2)	0.1533 (2)	0.0498 (7)	
H21A	0.4319	0.0589	0.1030	0.060*	
H21B	0.5479	0.0467	0.1618	0.060*	
C22	0.3696 (3)	0.0943 (3)	0.2571 (3)	0.0664 (9)	
H22A	0.4065	0.1135	0.3107	0.100*	
H22B	0.3589	0.0191	0.2752	0.100*	
H22C	0.2856	0.1506	0.2521	0.100*	
N6	0.05973 (18)	0.13511 (16)	0.50714 (14)	0.0260 (4)	
H7N	0.055 (2)	0.1145 (19)	0.4468 (11)	0.031*	
H8N	0.0105 (19)	0.1068 (19)	0.5573 (14)	0.031*	
C23	0.0145 (2)	0.26079 (18)	0.50850 (17)	0.0303 (5)	
H23A	0.0715	0.2928	0.4545	0.036*	
H23B	0.0200	0.2821	0.5764	0.036*	
C24	−0.1226 (2)	0.31081 (19)	0.48958 (17)	0.0330 (5)	
H24A	−0.1804	0.2829	0.5463	0.040*	
H24B	−0.1294	0.2849	0.4241	0.040*	
C25	−0.1664 (3)	0.4403 (2)	0.48336 (19)	0.0413 (6)	
H25A	−0.1697	0.4660	0.5517	0.050*	
H25B	−0.1023	0.4677	0.4328	0.050*	
C26	−0.2977 (3)	0.4922 (3)	0.4515 (3)	0.0643 (9)	
H26A	−0.2953	0.4664	0.3841	0.096*	
H26B	−0.3201	0.5750	0.4465	0.096*	
H26C	−0.3624	0.4686	0.5033	0.096*	
C27	0.1963 (2)	0.08328 (19)	0.52304 (17)	0.0305 (5)	
H27A	0.2048	0.1045	0.5900	0.037*	
H27B	0.2528	0.1141	0.4676	0.037*	
C28	0.2400 (2)	−0.04449 (19)	0.52239 (17)	0.0294 (5)	
H28A	0.2302	−0.0660	0.4559	0.035*	
H28B	0.1847	−0.0757	0.5787	0.035*	
C29	0.3800 (2)	−0.0954 (2)	0.53672 (19)	0.0369 (6)	
H29A	0.4354	−0.0679	0.4778	0.044*	
H29B	0.3906	−0.0690	0.6006	0.044*	
C30	0.4242 (3)	−0.2240 (2)	0.5439 (2)	0.0482 (7)	
H30A	0.3720	−0.2519	0.6038	0.072*	
H30B	0.5149	−0.2525	0.5517	0.072*	
H30C	0.4143	−0.2508	0.4807	0.072*	

### (II) Dibutylazanium 2-amino-4-nitrobenzoate Atomic displacement parameters (Å^2^)

**Table:**

	*U*^11^	*U*^22^	*U*^33^	*U*^12^	*U*^13^	*U*^23^
O1	0.0392 (9)	0.0268 (9)	0.0183 (7)	−0.0083 (7)	−0.0038 (6)	0.0024 (6)
O2	0.0368 (8)	0.0238 (8)	0.0190 (7)	−0.0068 (7)	−0.0059 (6)	−0.0027 (6)
O3	0.0605 (11)	0.0468 (11)	0.0205 (8)	−0.0248 (9)	−0.0028 (7)	−0.0085 (7)
O4	0.0737 (13)	0.0404 (11)	0.0232 (9)	−0.0241 (10)	−0.0038 (8)	0.0056 (8)
N1	0.0565 (13)	0.0220 (10)	0.0253 (10)	−0.0134 (9)	−0.0050 (9)	−0.0015 (8)
N2	0.0352 (10)	0.0366 (12)	0.0204 (10)	−0.0123 (9)	−0.0034 (8)	−0.0017 (9)
C1	0.0219 (10)	0.0246 (11)	0.0194 (10)	−0.0061 (8)	−0.0038 (8)	−0.0010 (8)
C2	0.0253 (10)	0.0224 (11)	0.0218 (11)	−0.0082 (9)	−0.0032 (8)	−0.0007 (8)
C3	0.0321 (11)	0.0243 (11)	0.0241 (11)	−0.0094 (9)	−0.0025 (9)	−0.0071 (9)
C4	0.0294 (11)	0.0277 (12)	0.0176 (10)	−0.0079 (9)	−0.0054 (8)	−0.0012 (9)
C5	0.0361 (12)	0.0234 (11)	0.0229 (11)	−0.0099 (9)	−0.0064 (9)	0.0022 (9)
C6	0.0332 (11)	0.0225 (11)	0.0208 (11)	−0.0082 (9)	−0.0040 (9)	−0.0028 (8)
C7	0.0248 (10)	0.0251 (12)	0.0209 (11)	−0.0069 (9)	−0.0068 (8)	0.0009 (9)
O5	0.0340 (8)	0.0267 (9)	0.0317 (9)	−0.0113 (7)	−0.0112 (7)	−0.0024 (7)
O6	0.0343 (8)	0.0322 (9)	0.0307 (9)	−0.0104 (7)	−0.0138 (7)	0.0031 (7)
O7	0.0389 (9)	0.0316 (9)	0.0345 (9)	−0.0113 (7)	−0.0176 (7)	0.0043 (7)
O8	0.0516 (10)	0.0308 (9)	0.0384 (9)	−0.0237 (8)	−0.0189 (8)	0.0031 (7)
N3	0.0343 (10)	0.0233 (10)	0.0285 (10)	−0.0123 (8)	−0.0110 (8)	0.0014 (8)
N4	0.0350 (10)	0.0281 (11)	0.0230 (9)	−0.0127 (8)	−0.0083 (8)	−0.0005 (8)
C8	0.0249 (10)	0.0223 (11)	0.0204 (10)	−0.0050 (9)	−0.0034 (8)	−0.0034 (8)
C9	0.0247 (10)	0.0206 (11)	0.0216 (10)	−0.0056 (8)	−0.0018 (8)	−0.0034 (8)
C10	0.0276 (11)	0.0207 (11)	0.0185 (10)	−0.0064 (9)	−0.0053 (8)	0.0000 (8)
C11	0.0284 (11)	0.0252 (11)	0.0203 (10)	−0.0094 (9)	−0.0063 (8)	−0.0029 (8)
C12	0.0346 (12)	0.0192 (11)	0.0305 (12)	−0.0097 (9)	−0.0099 (9)	0.0005 (9)
C13	0.0333 (12)	0.0227 (11)	0.0261 (11)	−0.0085 (9)	−0.0095 (9)	0.0026 (9)
C14	0.0237 (10)	0.0254 (12)	0.0241 (11)	−0.0042 (9)	−0.0048 (8)	−0.0029 (9)
N5	0.0304 (10)	0.0398 (12)	0.0293 (11)	−0.0113 (9)	−0.0111 (8)	0.0018 (9)
C15	0.0408 (14)	0.0484 (16)	0.0320 (13)	−0.0154 (12)	−0.0119 (11)	−0.0047 (11)
C16	0.0372 (13)	0.0428 (15)	0.0375 (14)	−0.0105 (11)	−0.0105 (11)	−0.0100 (11)
C17	0.103 (3)	0.0514 (19)	0.0531 (19)	−0.0208 (18)	−0.0289 (18)	−0.0123 (15)
C18	0.114 (3)	0.045 (2)	0.085 (3)	−0.016 (2)	−0.042 (2)	−0.0154 (18)
C19	0.0331 (12)	0.0408 (15)	0.0379 (14)	−0.0100 (11)	−0.0120 (10)	0.0109 (11)
C20	0.0487 (15)	0.0346 (14)	0.0399 (15)	−0.0121 (12)	−0.0049 (12)	−0.0001 (11)
C21	0.0435 (15)	0.0359 (15)	0.0654 (19)	−0.0098 (12)	−0.0071 (13)	0.0027 (13)
C22	0.0600 (19)	0.063 (2)	0.076 (2)	−0.0274 (17)	−0.0110 (17)	0.0130 (18)
N6	0.0338 (10)	0.0256 (10)	0.0184 (9)	−0.0090 (8)	−0.0051 (8)	−0.0005 (7)
C23	0.0439 (13)	0.0235 (12)	0.0239 (11)	−0.0107 (10)	−0.0067 (10)	−0.0013 (9)
C24	0.0423 (13)	0.0289 (13)	0.0234 (12)	−0.0066 (10)	−0.0031 (10)	−0.0014 (9)
C25	0.0599 (16)	0.0298 (13)	0.0283 (13)	−0.0031 (12)	−0.0093 (11)	−0.0072 (10)
C26	0.071 (2)	0.0424 (17)	0.063 (2)	0.0158 (15)	−0.0266 (17)	−0.0103 (15)
C27	0.0350 (12)	0.0330 (13)	0.0258 (12)	−0.0112 (10)	−0.0103 (9)	−0.0001 (9)
C28	0.0332 (12)	0.0324 (13)	0.0228 (11)	−0.0087 (10)	−0.0076 (9)	−0.0013 (9)
C29	0.0332 (12)	0.0445 (15)	0.0298 (13)	−0.0059 (11)	−0.0078 (10)	−0.0037 (11)
C30	0.0473 (15)	0.0453 (16)	0.0444 (16)	0.0044 (13)	−0.0187 (13)	−0.0083 (13)

### (II) Dibutylazanium 2-amino-4-nitrobenzoate Geometric parameters (Å, º)

**Table:**

O1—C7	1.262 (2)	C17—C18	1.494 (5)
O2—C7	1.267 (3)	C17—H17A	0.9900
O3—N2	1.228 (2)	C17—H17B	0.9900
O4—N2	1.225 (2)	C18—H18A	0.9800
N1—C2	1.360 (3)	C18—H18B	0.9800
N1—H1N	0.882 (10)	C18—H18C	0.9800
N1—H2N	0.882 (10)	C19—C20	1.502 (4)
N2—C4	1.477 (3)	C19—H19A	0.9900
C1—C6	1.403 (3)	C19—H19B	0.9900
C1—C2	1.420 (3)	C20—C21	1.535 (3)
C1—C7	1.509 (3)	C20—H20A	0.9900
C2—C3	1.413 (3)	C20—H20B	0.9900
C3—C4	1.375 (3)	C21—C22	1.526 (4)
C3—H3	0.9500	C21—H21A	0.9900
C4—C5	1.379 (3)	C21—H21B	0.9900
C5—C6	1.387 (3)	C22—H22A	0.9800
C5—H5	0.9500	C22—H22B	0.9800
C6—H6	0.9500	C22—H22C	0.9800
O5—C14	1.269 (3)	N6—C23	1.488 (3)
O6—C14	1.256 (3)	N6—C27	1.496 (3)
O7—N4	1.236 (2)	N6—H7N	0.888 (10)
O8—N4	1.231 (2)	N6—H8N	0.884 (10)
N3—C9	1.370 (3)	C23—C24	1.512 (3)
N3—H3N	0.885 (10)	C23—H23A	0.9900
N3—H4N	0.878 (10)	C23—H23B	0.9900
N4—C11	1.470 (3)	C24—C25	1.529 (3)
C8—C13	1.396 (3)	C24—H24A	0.9900
C8—C9	1.421 (3)	C24—H24B	0.9900
C8—C14	1.512 (3)	C25—C26	1.519 (4)
C9—C10	1.408 (3)	C25—H25A	0.9900
C10—C11	1.371 (3)	C25—H25B	0.9900
C10—H10	0.9500	C26—H26A	0.9800
C11—C12	1.385 (3)	C26—H26B	0.9800
C12—C13	1.387 (3)	C26—H26C	0.9800
C12—H12	0.9500	C27—C28	1.512 (3)
C13—H13	0.9500	C27—H27A	0.9900
N5—C15	1.490 (3)	C27—H27B	0.9900
N5—C19	1.494 (3)	C28—C29	1.525 (3)
N5—H5N	0.884 (10)	C28—H28A	0.9900
N5—H6N	0.891 (10)	C28—H28B	0.9900
C15—C16	1.505 (4)	C29—C30	1.518 (4)
C15—H15A	0.9900	C29—H29A	0.9900
C15—H15B	0.9900	C29—H29B	0.9900
C16—C17	1.507 (4)	C30—H30A	0.9800
C16—H16A	0.9900	C30—H30B	0.9800
C16—H16B	0.9900	C30—H30C	0.9800
			
C2—N1—H1N	111.8 (17)	C17—C18—H18C	109.5
C2—N1—H2N	118.2 (18)	H18A—C18—H18C	109.5
H1N—N1—H2N	123 (2)	H18B—C18—H18C	109.5
O4—N2—O3	123.54 (18)	N5—C19—C20	111.95 (19)
O4—N2—C4	118.01 (18)	N5—C19—H19A	109.2
O3—N2—C4	118.44 (18)	C20—C19—H19A	109.2
C6—C1—C2	119.04 (18)	N5—C19—H19B	109.2
C6—C1—C7	118.41 (18)	C20—C19—H19B	109.2
C2—C1—C7	122.55 (18)	H19A—C19—H19B	107.9
N1—C2—C3	118.25 (19)	C19—C20—C21	113.6 (2)
N1—C2—C1	123.47 (19)	C19—C20—H20A	108.8
C3—C2—C1	118.19 (18)	C21—C20—H20A	108.8
C4—C3—C2	119.68 (19)	C19—C20—H20B	108.8
C4—C3—H3	120.2	C21—C20—H20B	108.8
C2—C3—H3	120.2	H20A—C20—H20B	107.7
C3—C4—C5	123.59 (19)	C22—C21—C20	112.5 (3)
C3—C4—N2	117.74 (19)	C22—C21—H21A	109.1
C5—C4—N2	118.67 (19)	C20—C21—H21A	109.1
C4—C5—C6	116.9 (2)	C22—C21—H21B	109.1
C4—C5—H5	121.6	C20—C21—H21B	109.1
C6—C5—H5	121.6	H21A—C21—H21B	107.8
C5—C6—C1	122.5 (2)	C21—C22—H22A	109.5
C5—C6—H6	118.7	C21—C22—H22B	109.5
C1—C6—H6	118.7	H22A—C22—H22B	109.5
O1—C7—O2	124.31 (19)	C21—C22—H22C	109.5
O1—C7—C1	118.35 (19)	H22A—C22—H22C	109.5
O2—C7—C1	117.34 (18)	H22B—C22—H22C	109.5
C9—N3—H3N	114.1 (16)	C23—N6—C27	112.99 (17)
C9—N3—H4N	116.8 (15)	C23—N6—H7N	110.4 (16)
H3N—N3—H4N	120 (2)	C27—N6—H7N	107.3 (15)
O8—N4—O7	122.87 (17)	C23—N6—H8N	108.9 (16)
O8—N4—C11	118.69 (17)	C27—N6—H8N	108.0 (15)
O7—N4—C11	118.45 (17)	H7N—N6—H8N	109 (2)
C13—C8—C9	119.18 (18)	N6—C23—C24	111.70 (18)
C13—C8—C14	117.69 (18)	N6—C23—H23A	109.3
C9—C8—C14	123.04 (18)	C24—C23—H23A	109.3
N3—C9—C10	119.05 (18)	N6—C23—H23B	109.3
N3—C9—C8	123.08 (18)	C24—C23—H23B	109.3
C10—C9—C8	117.84 (18)	H23A—C23—H23B	107.9
C11—C10—C9	119.77 (19)	C23—C24—C25	111.90 (19)
C11—C10—H10	120.1	C23—C24—H24A	109.2
C9—C10—H10	120.1	C25—C24—H24A	109.2
C10—C11—C12	123.66 (19)	C23—C24—H24B	109.2
C10—C11—N4	117.78 (18)	C25—C24—H24B	109.2
C12—C11—N4	118.56 (18)	H24A—C24—H24B	107.9
C11—C12—C13	116.31 (19)	C26—C25—C24	112.6 (2)
C11—C12—H12	121.8	C26—C25—H25A	109.1
C13—C12—H12	121.8	C24—C25—H25A	109.1
C12—C13—C8	122.68 (19)	C26—C25—H25B	109.1
C12—C13—H13	118.7	C24—C25—H25B	109.1
C8—C13—H13	118.7	H25A—C25—H25B	107.8
O6—C14—O5	124.39 (19)	C25—C26—H26A	109.5
O6—C14—C8	116.94 (18)	C25—C26—H26B	109.5
O5—C14—C8	118.67 (18)	H26A—C26—H26B	109.5
C15—N5—C19	113.40 (19)	C25—C26—H26C	109.5
C15—N5—H5N	104.6 (17)	H26A—C26—H26C	109.5
C19—N5—H5N	107.4 (17)	H26B—C26—H26C	109.5
C15—N5—H6N	111.7 (17)	N6—C27—C28	112.16 (17)
C19—N5—H6N	108.7 (16)	N6—C27—H27A	109.2
H5N—N5—H6N	111 (2)	C28—C27—H27A	109.2
N5—C15—C16	112.20 (19)	N6—C27—H27B	109.2
N5—C15—H15A	109.2	C28—C27—H27B	109.2
C16—C15—H15A	109.2	H27A—C27—H27B	107.9
N5—C15—H15B	109.2	C27—C28—C29	111.31 (18)
C16—C15—H15B	109.2	C27—C28—H28A	109.4
H15A—C15—H15B	107.9	C29—C28—H28A	109.4
C15—C16—C17	111.6 (2)	C27—C28—H28B	109.4
C15—C16—H16A	109.3	C29—C28—H28B	109.4
C17—C16—H16A	109.3	H28A—C28—H28B	108.0
C15—C16—H16B	109.3	C30—C29—C28	112.7 (2)
C17—C16—H16B	109.3	C30—C29—H29A	109.1
H16A—C16—H16B	108.0	C28—C29—H29A	109.1
C18—C17—C16	113.9 (3)	C30—C29—H29B	109.1
C18—C17—H17A	108.8	C28—C29—H29B	109.1
C16—C17—H17A	108.8	H29A—C29—H29B	107.8
C18—C17—H17B	108.8	C29—C30—H30A	109.5
C16—C17—H17B	108.8	C29—C30—H30B	109.5
H17A—C17—H17B	107.7	H30A—C30—H30B	109.5
C17—C18—H18A	109.5	C29—C30—H30C	109.5
C17—C18—H18B	109.5	H30A—C30—H30C	109.5
H18A—C18—H18B	109.5	H30B—C30—H30C	109.5
			
C6—C1—C2—N1	−179.7 (2)	C9—C10—C11—C12	1.0 (3)
C7—C1—C2—N1	−0.8 (3)	C9—C10—C11—N4	−178.27 (18)
C6—C1—C2—C3	−3.2 (3)	O8—N4—C11—C10	−169.46 (19)
C7—C1—C2—C3	175.69 (18)	O7—N4—C11—C10	10.3 (3)
N1—C2—C3—C4	178.5 (2)	O8—N4—C11—C12	11.2 (3)
C1—C2—C3—C4	1.8 (3)	O7—N4—C11—C12	−169.0 (2)
C2—C3—C4—C5	1.2 (3)	C10—C11—C12—C13	−5.7 (3)
C2—C3—C4—N2	−178.83 (18)	N4—C11—C12—C13	173.56 (19)
O4—N2—C4—C3	−175.5 (2)	C11—C12—C13—C8	3.7 (3)
O3—N2—C4—C3	3.3 (3)	C9—C8—C13—C12	2.8 (3)
O4—N2—C4—C5	4.5 (3)	C14—C8—C13—C12	−173.8 (2)
O3—N2—C4—C5	−176.7 (2)	C13—C8—C14—O6	0.6 (3)
C3—C4—C5—C6	−2.6 (3)	C9—C8—C14—O6	−175.88 (19)
N2—C4—C5—C6	177.44 (18)	C13—C8—C14—O5	−179.63 (19)
C4—C5—C6—C1	1.0 (3)	C9—C8—C14—O5	3.9 (3)
C2—C1—C6—C5	1.9 (3)	C19—N5—C15—C16	−176.79 (19)
C7—C1—C6—C5	−177.09 (19)	N5—C15—C16—C17	−179.1 (2)
C6—C1—C7—O1	−168.45 (18)	C15—C16—C17—C18	171.9 (3)
C2—C1—C7—O1	12.6 (3)	C15—N5—C19—C20	171.3 (2)
C6—C1—C7—O2	11.6 (3)	N5—C19—C20—C21	−177.9 (2)
C2—C1—C7—O2	−167.31 (18)	C19—C20—C21—C22	49.5 (3)
C13—C8—C9—N3	170.5 (2)	C27—N6—C23—C24	178.58 (17)
C14—C8—C9—N3	−13.1 (3)	N6—C23—C24—C25	−176.00 (18)
C13—C8—C9—C10	−7.5 (3)	C23—C24—C25—C26	173.0 (2)
C14—C8—C9—C10	168.91 (19)	C23—N6—C27—C28	179.47 (18)
N3—C9—C10—C11	−172.40 (19)	N6—C27—C28—C29	178.96 (18)
C8—C9—C10—C11	5.7 (3)	C27—C28—C29—C30	176.0 (2)

### (II) Dibutylazanium 2-amino-4-nitrobenzoate Hydrogen-bond geometry (Å, º)

**Table:**

*D*—H···*A*	*D*—H	H···*A*	*D*···*A*	*D*—H···*A*
N1—H1*N*···O1	0.88 (1)	1.98 (2)	2.696 (2)	137 (2)
N1—H2*N*···O5^i^	0.88 (2)	2.38 (2)	3.226 (3)	160 (2)
N3—H3*N*···O5	0.88 (2)	2.01 (2)	2.714 (3)	136 (2)
N3—H4*N*···O2^ii^	0.88 (2)	2.19 (2)	3.052 (2)	168 (2)
N5—H5*N*···O5	0.88 (2)	1.89 (2)	2.757 (3)	166 (2)
N5—H6*N*···O6^iii^	0.89 (2)	1.81 (2)	2.697 (3)	173 (2)
N6—H7*N*···O2	0.89 (2)	1.89 (2)	2.759 (2)	167 (2)
N6—H8*N*···O1^iv^	0.89 (2)	1.85 (2)	2.712 (2)	163 (2)
C19—H19*A*···O7^v^	0.99	2.56	3.343 (3)	136
C20—H20*B*···O6^iii^	0.99	2.56	3.297 (3)	131
C27—H27*A*···O4^vi^	0.99	2.57	3.550 (3)	169

Symmetry codes: (i) −*x*+1, −*y*, −*z*; (ii) *x*+1, *y*, *z*; (iii) −*x*+1, −*y*+1, −*z*; (iv) −*x*, −*y*, −*z*+1; (v) *x*−1, *y*, *z*; (vi) *x*, *y*, *z*+1.
